# ARL3 and ARL13B GTPases participate in distinct steps of INPP5E targeting to the ciliary membrane

**DOI:** 10.1242/bio.058843

**Published:** 2021-09-28

**Authors:** Sayaka Fujisawa, Hantian Qiu, Shohei Nozaki, Shuhei Chiba, Yohei Katoh, Kazuhisa Nakayama

**Affiliations:** 1 Department of Physiological Chemistry, Graduate School of Pharmaceutical Sciences, Kyoto University, Sakyo-ku, Kyoto 606-8501, Japan; 2 Department of Genetic Disease Research, Graduate School of Medicine, Osaka City University, Abeno-ku, Osaka 545-8585, Japan

**Keywords:** ARL3, ARL13B, INPP5E, Cilia

## Abstract

INPP5E, a phosphoinositide 5-phosphatase, localizes on the ciliary membrane via its C-terminal prenyl moiety, and maintains the distinct ciliary phosphoinositide composition. The ARL3 GTPase contributes to the ciliary membrane localization of INPP5E by stimulating the release of PDE6D bound to prenylated INPP5E. Another GTPase, ARL13B, which is localized on the ciliary membrane, contributes to the ciliary membrane retention of INPP5E by directly binding to its ciliary targeting sequence. However, as ARL13B was shown to act as a guanine nucleotide exchange factor (GEF) for ARL3, it is also possible that ARL13B indirectly mediates the ciliary INPP5E localization via activating ARL3. We here show that INPP5E is delocalized from cilia in both *ARL3*-knockout (KO) and *ARL13B*-KO cells. However, some of the abnormal phenotypes were different between these KO cells, while others were found to be common, indicating the parallel roles of ARL3 and ARL13B, at least concerning some cellular functions. For several variants of ARL13B, their ability to interact with INPP5E, rather than their ability as an ARL3-GEF, was associated with whether they could rescue the ciliary localization of INPP5E in *ARL13B*-KO cells. These observations together indicate that ARL13B determines the ciliary localization of INPP5E, mainly by its direct binding to INPP5E.

## Abstract (precis)

**Summary:** This paper investigates the role of ARL13B in targeting of INPP5E to the ciliary membrane, and shows the direct role of ARL13B within cilia, independently of its ARL3-GEF activity.

## INTRODUCTION

ARF/ARL and RAB families of small GTPases function as molecular switches by cycling between a GDP-bound inactive state and a GTP-bound active state to regulate various cellular processes, such as membrane trafficking. Small GTPases belonging to these two families also participate in ciliogenesis and ciliary protein trafficking; these include ARF4, ARL3, ARL6/BBS3, ARL13B, RAB8, RAB10, RAB23, and RAB28 (Fisher et al., 2020; Lim et al., 2011; Zhang et al., 2013). In addition, RAB-like GTPases, including RABL2, RABL4/IFT27/BBS19, and RABL5/IFT22, are also associated with ciliary functions (Dateyama et al., 2019; Kanie et al., 2017; Nakayama and Katoh, 2018; Nishijima et al., 2017; Taschner and Lorentzen, 2016).

Primary cilia are microtubule-based protrusions on the surface of various eukaryotic cells, which act as cellular sensors of extracellular mechanical stimuli, such as fluid flow, and of signaling molecules, such as the Hedgehog (Hh) family of morphogens (Bangs and Anderson, 2017; Gigante and Caspary, 2020). To achieve these sensory functions, specific proteins, such as G protein-coupled receptors (GPCRs) and ion channels, are located on the ciliary membrane and in the cilioplasm (Ishikawa et al., 2012; Mick et al., 2015). The distinction between cilia and the cell body is dependent on the ciliary gate, which is composed of transition fibers of the basal body and the transition zone (TZ), which is a specialized area located at the base of cilia (Garcia-Gonzalo and Reiter, 2017; Gonçalves and Pelletier, 2017). The TZ acts as a permeability/diffusion barrier that restricts the ciliary entry and exit of soluble and membrane proteins (Jensen and Leroux, 2017; Nachury and Mick, 2019; Stephen and Ismail, 2016). Analyses using super-resolution microscopy demonstrated the structural framework of the TZ (Katoh et al., 2020; Shi et al., 2017; Yang et al., 2015). The importance of these structures has been indicated by the fact that mutations in the genes encoding TZ components cause a broad spectrum of congenital disorders, generally called the ciliopathies (Braun and Hildebrandt, 2017; Reiter and Leroux, 2017); these include Meckel syndrome, Joubert syndrome (JBTS), Bardet-Biedl syndrome (BBS), and nephronophthisis.

The bidirectional trafficking of proteins within cilia and their entry and exit across the TZ are mediated by the intraflagellar transport (IFT) machinery (Ishikawa and Marshall, 2011; Nakayama and Katoh, 2020; Taschner and Lorentzen, 2016), which comprises large multisubunit complexes. The IFT-B complex is composed of 16 subunits, including IFT27/RABL4 and IFT22/RABL5, and mediates anterograde protein trafficking together with the kinesin-2 motor. The IFT-A complex, which is composed of six subunits plus the TULP3 adaptor protein, mediates not only retrograde trafficking with the aid of the dynein-2 motor complex, but also participates in the import of ciliary membrane proteins across the ciliary gate (Badgandi et al., 2017; Han et al., 2019; Hirano et al., 2017; Kobayashi et al., 2021; Mukhopadhyay et al., 2010; Park et al., 2013; Picariello et al., 2019). In addition, the BBSome complex composed of eight BBS proteins plus the ARL6/BBS3 GTPase acts as an adaptor between the IFT machinery and ciliary transmembrane proteins, and mediates the export of ciliary membrane proteins (Eguether et al., 2014; Lechtreck et al., 2013; Liew et al., 2014; Liu and Lechtreck, 2018; Nozaki et al., 2018; 2019; Ye et al., 2018).

On the other hand, ciliary membrane targeting of lipid-anchored proteins is mediated by a distinct system (Jensen and Leroux, 2017; Stephen and Ismail, 2016). After their synthesis and lipid modification, C-terminally prenylated and N-terminally myristoylated membrane proteins are first trapped in the cytosol by PDE6D and UNC119, respectively, both of which are RhoGDI-like solubilizing factors for lipidated proteins (Stephen and Ismail, 2016). These proteins are then released from PDE6D or UNC119 by allosteric binding of the ARL3 GTPase to the solubilizing factor (Ismail et al., 2011).

INPP5E is a phosphoinositide 5-phosphatase with a prenyl moiety covalently attached to its C-terminus, and is involved in the maintenance of the distinct phosphoinositide composition on the ciliary membrane, i.e., a high PtdIns(4)P level and a low PtdIns(4,5)P_2_ level (Chávez et al., 2015; Conduit and Vanhaesebroeck, 2020; Garcia-Gonzalo et al., 2015; Nakatsu, 2015). This PtdIns(4)P-rich condition in the cilia participates in the regulation of TULP3 function. As the TULP3 Tubby domain binds to PtdIns(4,5)P_2_, but not to PtdIns(4)P, retrograde trafficking of ciliary membrane proteins, such as GPR161, mediated by the TULP3 adaptor protein together with the IFT-A complex, is impaired under ciliary PtdIns(4,5)P_2_-rich conditions in the absence of INPP5E (Chávez et al., 2015; Garcia-Gonzalo et al., 2015). GPR161 is a ciliary GPCR that negatively regulates Hh signaling under basal conditions, and exits cilia when the Hh pathway is activated (Mukhopadhyay and Rohatgi, 2014; Nachury and Mick, 2019).

As described above, ARL3, together with PDE6D, regulates the ciliary membrane localization of C-terminally prenylated INPP5E (Fansa et al., 2016; Humbert et al., 2012). In addition, another small GTPase, ARL13B, also contributes to the steady-state localization of INPP5E on the ciliary membrane. Two possible roles of ARL13B in INPP5E localization were proposed, although these are not mutually exclusive. One is that ARL13B directly participates in the ciliary membrane retention of INPP5E by binding to its ciliary targeting sequence (CTS), F^609^DRELYL^615^, in the C-terminal region (Humbert et al., 2012). On the other hand, ARL13B was reported to act as a guanine nucleotide exchange factor (GEF) for ARL3 (Alkanderi et al., 2018; El Maghloob et al., 2021; Gotthardt et al., 2015; Ivanova et al., 2017; Zhang et al., 2016), and has been proposed to indirectly participate in the ciliary targeting of INPP5E via promoting its release from PDE6D with the aid of activated ARL3 (Stephen and Ismail, 2016). In this context, it is noteworthy that mutations of not only *INPP5E*/*JBTS1* itself (Bielas et al., 2009; Jacoby et al., 2009) but also *ARL13B*/*JBTS8* (Cantagrel et al., 2008), *PDE6D*/*JBTS22* (Thomas et al., 2014), and *ARL3*/*JBTS35* (Alkanderi et al., 2018) are found in individuals with JBTS (Braun and Hildebrandt, 2017; Parisi and Glass, 2017).

By comparing the phenotypes of KO cells of *INPP5E* and *ARL13B* that were established from human telomerase reverse transcriptase-immortalized retinal pigment epithelial 1 (hTERT-RPE1) cells, and those of *INPP5E*-KO cells exogenously expressing INPP5E mutants, we recently showed that the direct binding of ARL13B to the CTS of INPP5E is essential for the steady-state localization of INPP5E on the ciliary membrane, but is dispensable for its entry into cilia (Qiu et al., 2021). To investigate the roles of ARL3 and ARL13B in the ciliary membrane targeting of INPP5E in more detail, we here established *ARL3*-KO hTERT-RPE1 cells and compared their phenotypes with those of *ARL13B*-KO and *INPP5E*-KO cells with the same cell background.

## RESULTS

### Mutually exclusive binding of ARL3 and INPP5E to PDE6D, and that of PDE6D and ARL13B to INPP5E

At the beginning of this study, we systematically analyzed the interactions among ARL3, PDE6D, INPP5E, and ARL13B, although these interactions have been analyzed individually in several previous studies (Fansa et al., 2016; Humbert et al., 2012; Thomas et al., 2014; Van Valkenburgh et al., 2001; Wätzlich et al., 2013); their interactions have not been considered in detail. For this purpose, we used the visible immunoprecipitation (VIP) assay, followed by conventional immunoblotting analysis. The VIP assay is a variation of the coimmunoprecipitation assay using fluorescent fusion proteins expressed in cells, which hence enables the visual detection of protein–protein interactions under a fluorescence microscope (Katoh et al., 2015, 2016).

**Fig. 1.**
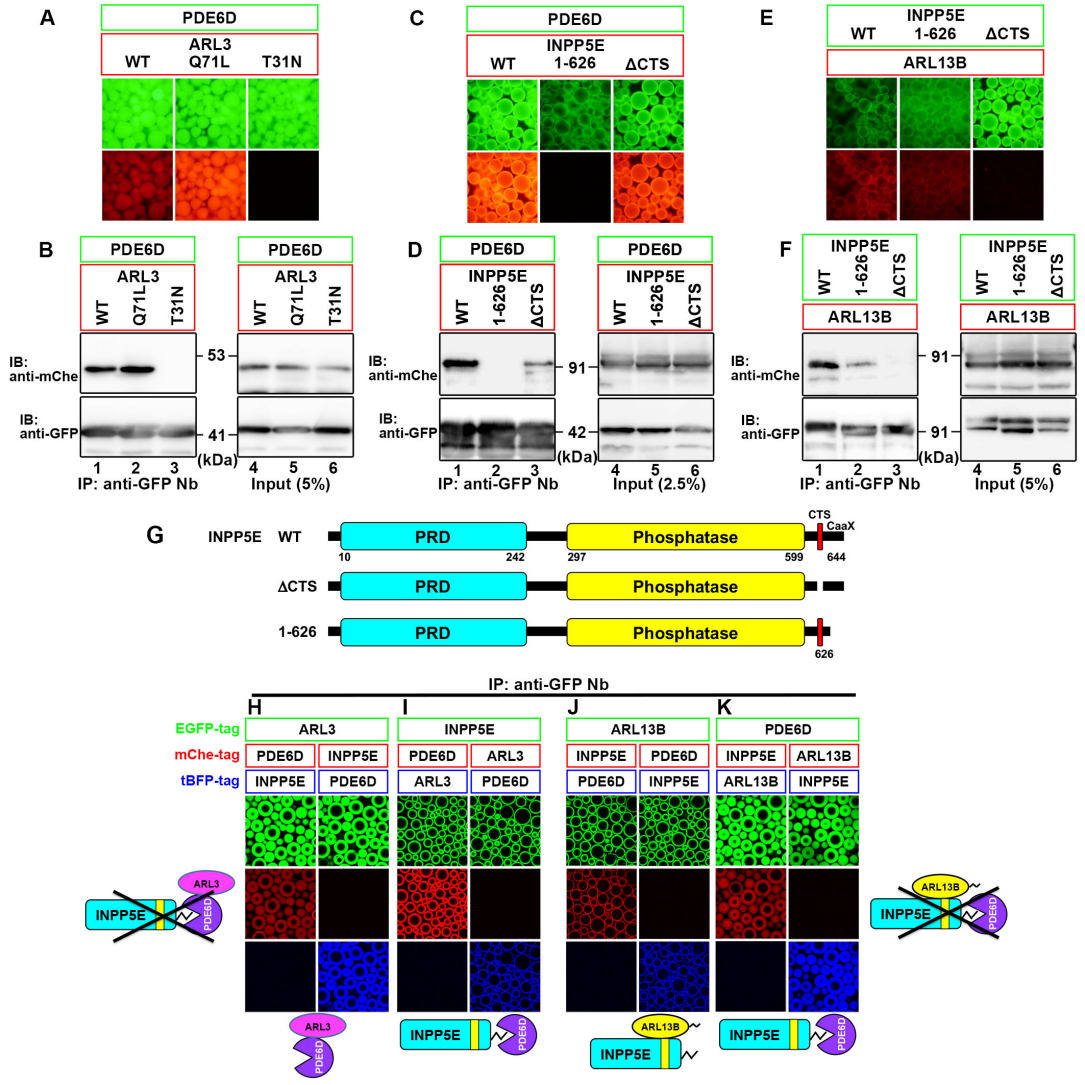
**Mutually exclusive binding of ARL3 and INPP5E to PDE6D, and that of PDE6D and ARL13B to INPP5E.** (A–F) HEK293T cells were cotransfected with expression vectors for EGFP-PDE6D and an mChe-fused ARL3 construct as indicated (A, B), for EGFP-PDE6D and an mChe-fused INPP5E construct as indicated (C, D), or for ARL13B-mChe and EGFP-fused INPP5E construct as indicated (E, F). Lysates prepared from the transfected cells were subjected to immunoprecipitation with GST-fused anti-GFP Nb prebound to glutathione-Sepharose beads, and the precipitated beads bearing fluorescent fusion proteins were observed under a microscope (A, C, E). Proteins bound to the beads were then subjected to SDS-PAGE, followed by immunoblotting analysis using anti-mChe and anti-GFP antibodies (B, D, F). (G) Schematic representation of the domain organization of INPP5E and its mutants used in this study. PRD, proline-rich domain. (H–K) Lysates were prepared from cells cotransfected with expression vectors for the indicated combinations of EGFP-fused, mChe-fused, and tBFP-fused constructs; to express approximately equal amount of the ARL3, INPP5E, PDE6D, and ARL13B proteins fused with EGFP, mChe, or tBFP, HEK293T cells were transfected with the expression vectors for ARL3, INPP5E, and PDE6D at the ratio of 1:6:1 (H, I), or those for ARL13B, INPP5E and PDE6D at the ratio of 4:5:1 (J, K). The lysates were then immunoprecipitated with GST–anti-GFP Nb, followed by observation under a microscope. Expected interactions are shown schematically.

Lysates prepared from HEK293T cells coexpressing EGFP-PDE6D and various mCherry (mChe)-fused ARL3 constructs were subjected to immunoprecipitation using a GST-fused anti-GFP nanobody (Nb) prebound to glutathione-Sepharose beads, and the precipitated beads were observed under a fluorescence microscope; red signals were observed in cells expressing mChe-fused wild-type (WT) ARL3 and its GTP-locked mutant (Q71L), but not its GDP-locked mutant (T31N) (Fig. 1A). The VIP results were confirmed by subsequent immunoblotting analysis. Bands for mChe-fused ARL3(WT) and ARL3(Q71L), but not ARL3(T31N), were detected (Fig. 1B), supporting the idea that ARL3 binds to PDE6D in its GTP-bound state. As shown in Fig. 1C and D, EGFP-PDE6D coimmunoprecipitated mChe-fused INPP5E(WT), but not its C-terminal deletion mutant, INPP5E(1-626) (Fig. 1G), which was first found in a family with MORM syndrome and lacks the prenylation site (Jacoby et al., 2009). As shown in Fig. 1E and F, ARL13B-mChe was coimmunoprecipitated with EGFP-fused INPP5E(WT) but not with INPP5E(ΔCTS), which lacks the CTS (F^609^DRELYL^615^; Fig. 1G) (Humbert et al., 2012), confirming that ARL13B binds to INPP5E by recognizing its CTS. INPP5E(1-626) retained the ability to interact with ARL13B, indicating that ARL13B can bind to INPP5E independently of its prenylation.

C-terminally prenylated INPP5E trapped by PDE6D in the cytosol was shown to be released by the allosteric binding of ARL3 to PDE6D, and then became anchored to the membrane (Stephen and Ismail, 2016). To confirm this notion, we performed the VIP assay using ARL3, INPP5E, and PDE6D fused to EGFP, mChe, or TagBFP (tBFP). When lysates of HEK293T cells coexpressing ARL3-EGFP with mChe-INPP5E and tBFP-PDE6D or mChe-PDE6D and tBFP-INPP5E were subjected to the VIP assay using GST-tagged anti-GFP Nb, only mChe-fused or tBFP-fused PDE6D was coimmunoprecipitated with ARL3-EGFP (Fig. 1H). Reciprocally, EGFP-INPP5E coprecipitated mChe/tBFP-fused PDE6D but not ARL3 (Fig. 1I). These results are consistent with the notion that ARL3 acts as an allosteric releasing factor for PDE6D from INPP5E.

We then investigated whether ARL13B can bind the INPP5E sequestered by PDE6D. As shown in Fig. 1J, ARL13B-EGFP coprecipitated mChe/tBFP-fused INPP5E but not PDE6D. Reciprocally, EGFP-PDE6D coprecipitated mChe/tBFP-fused INPP5E but not ARL13B (Fig. 1K). These results exclude the possibility that ARL13B, INPP5E, and PDE6D form a tripartite complex. Altogether, these results support that ARL13B can bind to INPP5E after its ARL3-promoted release from PDE6D.

### Similar and distinct phenotypes of *ARL3*-KO, *ARL13B*-KO, and *INPP5E*-KO cells

As described in the Introduction, our previous studies using *ARL13B*-KO cells (Nozaki et al., 2017) and *INPP5E*-KO cells (Qiu et al., 2021) established from hTERT-RPE1 cells showed that ARL13B targets INPP5E to the ciliary membrane by directly binding its CTS. On the other hand, it is also possible that ARL13B is indirectly involved in the ciliary membrane targeting of INPP5E, by acting as a GEF for ARL3; namely, via promoting the release of PDE6D from INPP5E by activating ARL3 as a releasing factor (Gotthardt et al., 2015; Stephen and Ismail, 2016).

Hu and colleagues performed a study using *Caenorhabditis elegans* mutants, and suggested the involvement of ARL-13 and ARL-3 in IFT and ciliogenesis about 10 years ago (Li et al., 2010). They then demonstrated mainly by *in vitro* experiments that *C. elegans* ARL-3 is activated by ARL-13 (Zhang et al., 2016), after the report that mammalian ARL13B acts as a GEF for ARL3 (Gotthardt et al., 2015). On the other hand, although *Arl3*-KO mice were established in 2006 and photoreceptor-specific *Arl3*-KO mice were established more recently, the cellular functions of ARL3 have not been thoroughly investigated using cells derived from these KO mice (Hanke-Gogokhia et al., 2016; Schrick et al., 2006). In particular, in photoreceptor cells of *Arl3*-KO mice, INPP5E localizes to the inner segments and is absent from the outer segments (Hanke-Gogokhia et al., 2016), which is equivalent to cilia of normal cells. Furthermore, in a previous study using siRNA against Arl3 (Fansa et al., 2016), although exogenously expressed INPP5E demonstrated a tendency to be delocalized from cilia by Arl3 siRNA treatment, the difference in ciliary INPP5E localization between control and Arl3 siRNA-treated cells was not statistically significant (Fansa et al., 2016). We therefore established *ARL3*-KO cell lines from hTERT-RPE1 cells to directly compare their phenotypes with those of *ARL13B*-KO and *INPP5E*-KO cells with the same cell background.

**Fig. 2.**
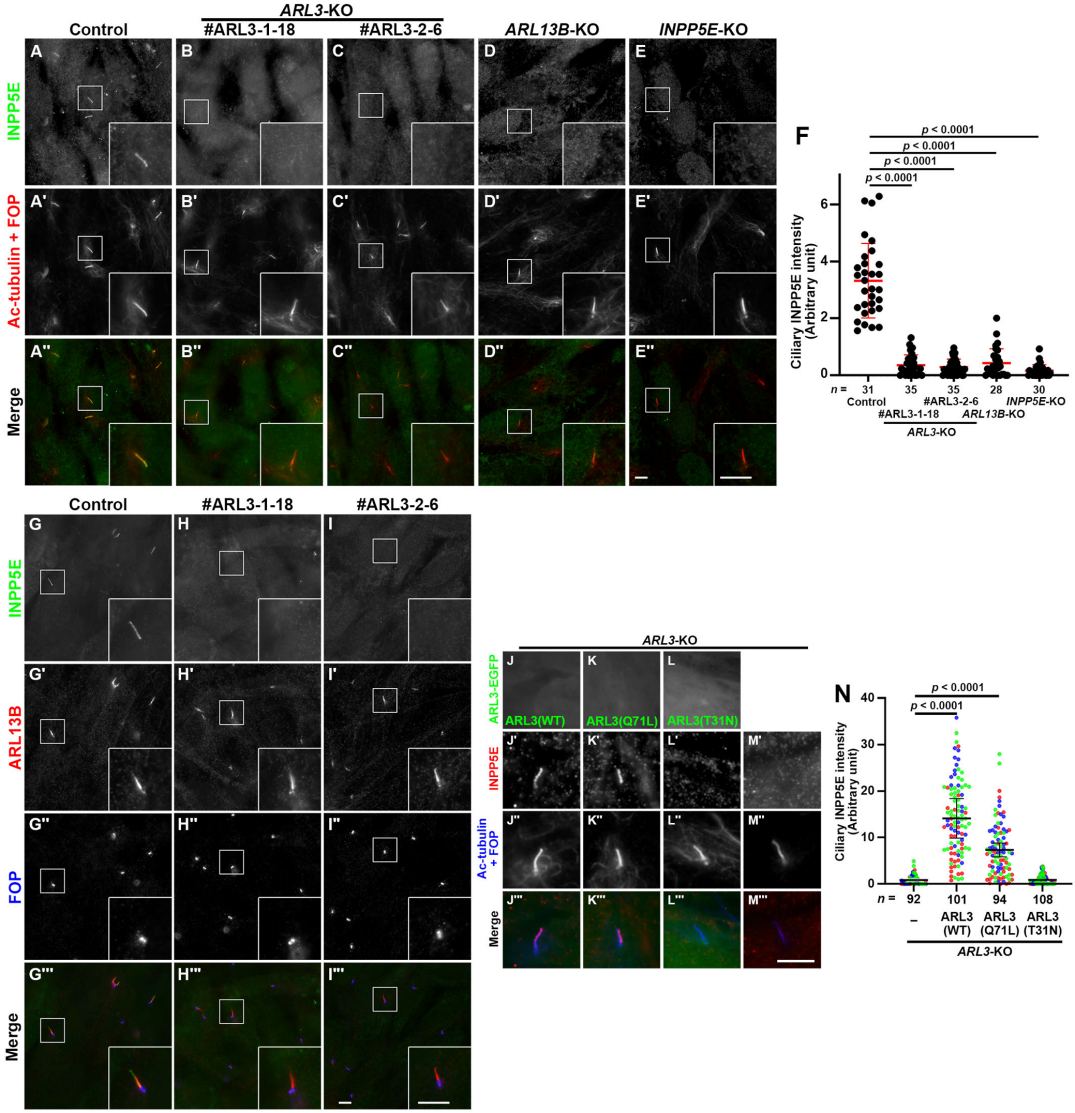
**Delocalization of INPP5E from the ciliary membrane in *ARL3*-KO, *ARL13B*-KO, and *INPP5E*-KO cells.** Control RPE1 cells (A), the *ARL3*-KO cell lines #ARL3-1-18 (B) and #ARL3-2-6 (C), the *ARL13B*-KO cell line #ARL13B-1-2 (D), and the *INPP5E*-KO cell line #INPP5E-2-2 (E) were serum-starved for 24 h to induce ciliogenesis, and immunostained for INPP5E (A–E) and Ac-tubulin+FOP (A′–E′). Insets show 2.5-fold magnified images of the boxed regions. (F) Relative ciliary staining intensities of INPP5E in control, *ARL3*-KO, *ARL13B*-KO, and *INPP5E*-KO cells were estimated and expressed as scatter plots. The total number of cells analyzed (*n*) are indicated. Horizontal lines are means, and error bars indicate the SD. Statistical significances were calculated using one-way ANOVA followed by the Dunnett's multiple comparison test. (G–I) Control RPE1 cells (G) and the *ARL3*-KO cell lines #ARL3-1-18 (H) and #ARL3-2-6 (I) were serum-starved for 24 h and triply immunostained for INPP5E, ARL13B, and FOP. Insets show 2.5-fold magnified images of the boxed regions. (J–M) *ARL3*-KO cells (#ARL3-1-18), which stably express EGFP-fused ARL3(WT) (J), ARL3(Q71L) (K), or ARL3(T31N) (L), were serum starved for 24 h and triply immunostained for GFP, INPP5E, and Ac-tubulin+FOP. Scale bars, 5 µm. (N) Relative ciliary staining intensities of INPP5E in individual cells shown in (J′)–(M′) were estimated and expressed as scatter plots. Different colored dots represent three independent experiments; the horizontal lines indicate the means, and error bars are the SDs. Statistical significances were calculated using one-way ANOVA followed by the Dunnett's multiple comparison test.

Two independent KO cell lines (#ARL3-1-18 and #ARL3-2-6) that were established using distinct target sequences were selected for the following analyses (Fig. S1). INPP5E was not detectable within the cilia of these cells, similarly to *ARL13B*-KO and *INPP5E*-KO cells (Fig. 2A–F), demonstrating the ARL3-dependent localization of INPP5E on the ciliary membrane. On the other hand, ARL13B was retained on the ciliary membrane of these *ARL3*-KO cells (Fig. 2G–I), excluding the possibility that the delocalization of INPP5E in the absence of ARL3 was a result of ARL13B delocalization.

To rule out the possibility that the delocalization of INPP5E from the ciliary membrane observed in *ARL3*-KO cells resulted from off-target effects inherent to the CRISPR/Cas9 system, we next investigated whether the exogenous expression of ARL3 is able to restore the localization of INPP5E on the ciliary membrane. As shown in Fig. 2J–N, the stable expression of EGFP-fused ARL3(WT) and its GTP-locked mutant, ARL3(Q71L), but not its GDP-locked mutant, ARL3(T31N), in the *ARL3*-KO cell line (#ARL3-2-6) restored the localization of INPP5E on the ciliary membrane, indicating that GTP-bound ARL3 participates in INPP5E localization, likely via promoting the release of PDE6D from INPP5E (Fig. 1).

**Fig. 3.**
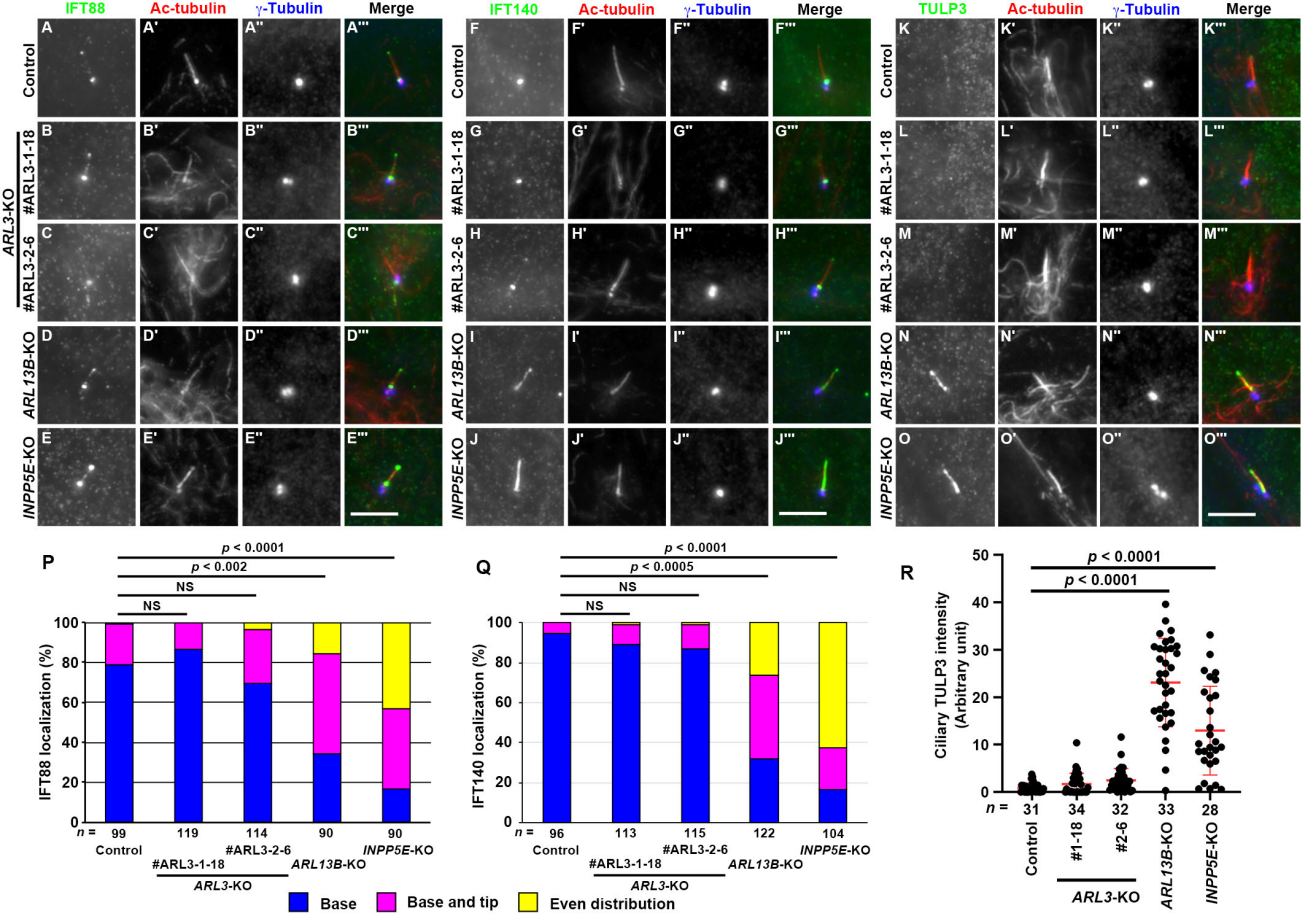
**Localizations of IFT-B and IFT-A proteins are differentially affected in *ARL3*-KO, *ARL13B*-KO, and *INPP5E*-KO cells.** (A–O) Control RPE1 cells (A, F, K), the *ARL3*-KO cell lines #ARL3-1-18 (B, G, L) and #ARL3-2-6 (C, H, M), the *ARL13B*-KO cell line #ARL13B-1-2 (D, I, N), and the *INPP5E*-KO cell line #INPP5E-2-2 (E, J, O) were serum-starved for 24 h and triply immunostained with antibodies against IFT88 (A–E), IFT140 (F–J), or TULP3 (K–O), Ac-tubulin (A′–O′), and γ-tubulin (A″–O″). Scale bars, 5 µm. (P, Q) Localization of IFT88 (P) and IFT140 (Q) in control, *ARL3*-KO, *ARL13B*-KO, and *INPP5E*-KO cells was classified as ‘localization to ciliary base’, ‘localization to ciliary base and tip’, and ‘even distribution throughout cilia’, and the number of cells in each category was counted. The percentages of these populations are expressed as stacked bar graphs. Values are shown as the means of three independent experiments, and the total number of cells analyzed (*n*) are indicated. In each set of experiments, 26 to 47 cells (P) and 30 to 46 cells (Q) were analyzed. Statistical significances were calculated for the ‘base’ category using one-way ANOVA followed by the Tukey–Kramer test. (R) Relative ciliary staining intensities of TULP3 in control, *ARL3*-KO, *ARL13B*-KO, and *INPP5E*-KO cells were estimated and expressed as scatter plots. The horizontal lines indicate the means, and the error bars are the SDs. Statistical significances were calculated using one-way ANOVA followed by the Dunnett's multiple comparison test.

We then compared the localization of components of the IFT machinery between *ARL3*-KO cells and *ARL13B*-KO and *INPP5E*-KO cells, because our previous studies demonstrated that a larger amount of IFT-A and IFT-B proteins were accumulated within cilia in *ARL13B*-KO and *INPP5E*-KO cells compared with control RPE1 cells (Nozaki et al., 2017; Qiu et al., 2021). As shown in Fig. 3, the IFT-B subunit IFT88 (Fig. 3, compare D and E with A; also see Fig. 3P), and the IFT-A subunit IFT140 (Fig. 3, compare I and J with F; also see Fig. 3Q) were significantly enriched within cilia in *ARL13B*-KO and *INPP5E*-KO cells compared with their predominant localization to the ciliary base in control RPE1 cells, particularly around the transition fibers and in the TZ (Ishida et al., 2021; Katoh et al., 2020; Yang et al., 2015). By contrast, the predominant localization to the ciliary base of IFT88 and IFT140 was not significantly altered in *ARL3*-KO cells compared with control cells (Fig. 3A–C and F–H, respectively; also see Fig. 3P and Q).

These observations were somewhat unexpected, given that in the absence of ciliary INPP5E, PtdIns(4,5)P_2_ is enriched on the ciliary membrane (Chávez et al., 2015; Garcia-Gonzalo et al., 2015; Nakatsu, 2015), and thereby TULP3, which is a PtdIns(4,5)P_2_-binding protein that acts as an adaptor between the IFT-A complex and the ciliary membrane (Mukhopadhyay et al., 2010), is retained on the ciliary membrane (Chávez et al., 2015; Garcia-Gonzalo et al., 2015). We therefore compared the localization of endogenous TULP3 in *ARL3*-KO, *ARL13B*-KO, and *INPP5E*-KO cells; in our previous studies, we showed the significant enrichment of exogenously expressed EGFP-TULP3 within cilia in *ARL13B*-KO and *INPP5E*-KO cells (Nozaki et al., 2017; Qiu et al., 2021). Using a polyclonal antibody against TULP3, endogenous TULP3 was not detected within cilia or was barely detectable at the ciliary base in control RPE1 cells (Fig. 3K). In striking contrast, TULP3 was clearly enriched throughout cilia in *ARL13B*-KO and *INPP5E*-KO cells (Fig. 3N and O; also see Fig. 3R), which was in agreement with our previous studies (Nozaki et al., 2017; Qiu et al., 2021). However, in *ARL3*-KO cells, TULP3 was not detectable within cilia (Fig. 3L and M; also see Fig. 3R), even though INPP5E was not found in the cilia of these cells, as in *ARL13B*-KO and *INPP5E*-KO cells (Fig. 2) (see Discussion).

**Fig. 4.**
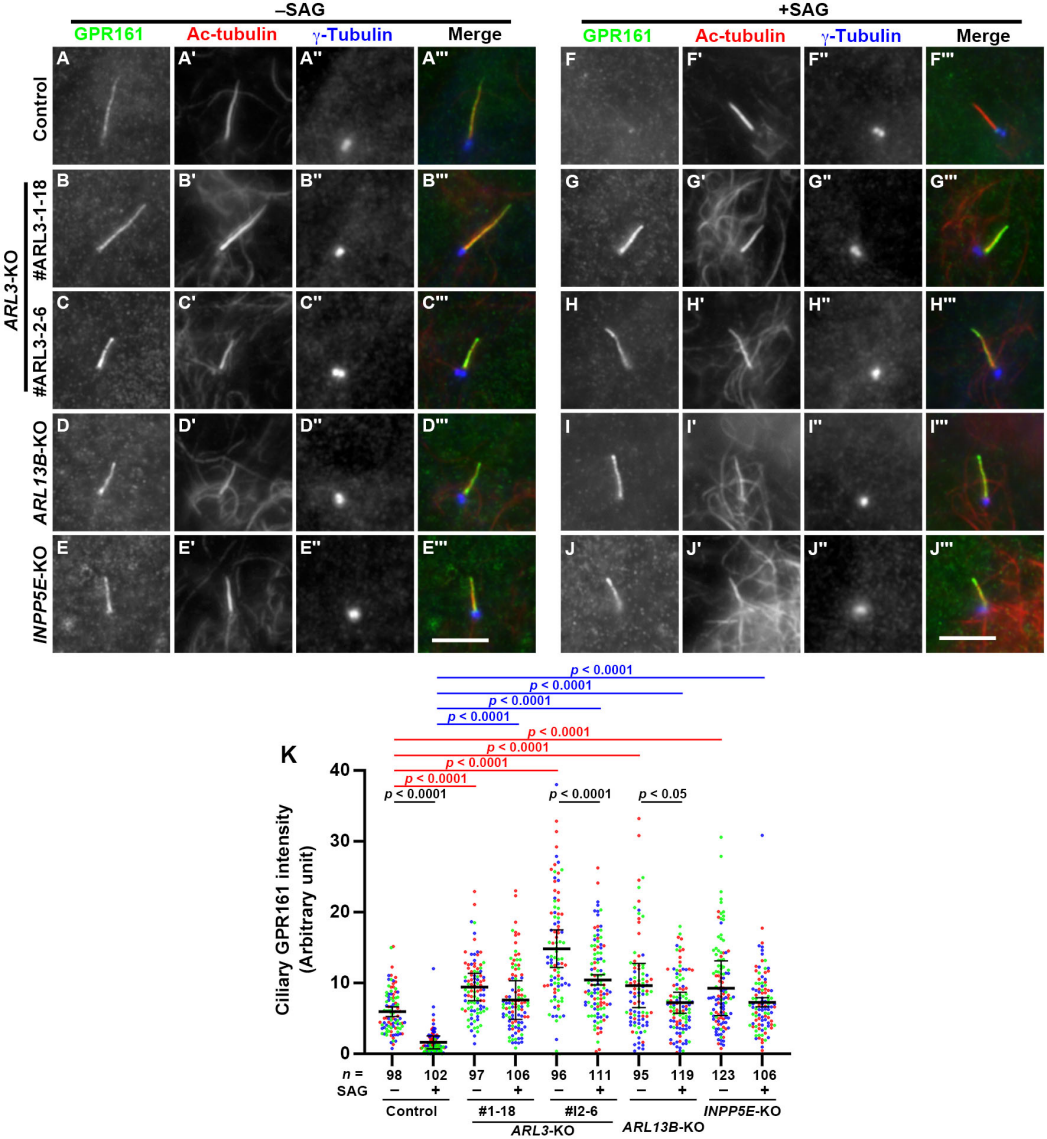
**Stimulated exit of GPR161 from cilia is blocked in *ARL3*-KO, *ARL13B*-KO, and *INPP5E*-KO cells.** Control RPE1 cells (A, F), the *ARL3*-KO cell lines #ARL3-1-18 (B, G) and #ARL3-2-6 (C, H), the *ARL13B*-KO cell line #ARL13B-1-2 (D, I), and the *INPP5E*-KO cell line #INPP5E-2-2 (E, J) were serum-starved for 24 h, and then cultured in the absence (A–E; –SAG) or presence (F–J; +SAG) of 200 nM SAG for a further 24 h, and triply immunostained for GPR161 (A–J), Ac-tubulin (A′–J′), and γ-tubulin (A″–J″). Scale bars, 5 µm. (K) Relative ciliary staining intensities of GPR161 in control, *ARL3*-KO, *ARL13B*-KO, and *INPP5E*-KO cells under basal and SAG-stimulated conditions are represented as scatter plots. Different colored dots represent three independent experiments, horizontal lines indicate the means, and the error bars are the SDs. Statistical significances were calculated using one-way ANOVA followed by the Tukey-Kramer test.

We then analyzed the localization of GPR161 under basal and Hh pathway-stimulated conditions, because we have previously shown that GRP161 does not exit cilia in *ARL13B*-KO and *INPP5E*-KO RPE1 cells when cells are stimulated with Smoothened agonist (SAG) (Nozaki et al., 2017; Qiu et al., 2021). Upon SAG treatment, GPR161 exited cilia in control RPE1 cells (Fig. 4, compare F with A), but was retained within cilia in *ARL13B*-KO and *INPP5E*-KO cells (Fig. 4, compare I and J with D and E; also see Fig. 4K). In *ARL3*-KO cells, the results were essentially the same as those in *ARL13B*-KO and *INPP5E*-KO cells; i.e., compared with control RPE1 cells (Fig. 4A and F), the basal level of ciliary GPR161 was significantly increased (Fig. 4B and C; also see Fig. 4K), and a high level of GPR161 was retained within cilia even upon their stimulation with SAG (Fig. 4, compare G and H with B and C; also see Fig. 4K). On the other hand, the ciliary levels of Smoothened (SMO) under basal and SAG-stimulated conditions in *ARL3*-KO cells were similar to those observed in control RPE1 cells; SMO was under the detection level within cilia without SAG treatment (Fig. S2A–C), whereas it became detectable within cilia when control cells and *ARL3*-KO cells were stimulated with SAG (Fig. S2D–F). These observations of SMO localization in *ARL3*-KO cells under basal and SAG-stimulated conditions were essentially the same as those observed in *ARL13B*-KO and *INPP5E*-KO cells, as reported previously (Qiu et al., 2021).

### Absence of the BBSome and ARL6 in the cilia of *ARL3*-KO, *ARL13B*-KO, and *INPP5E*-KO cells

**Fig. 5.**
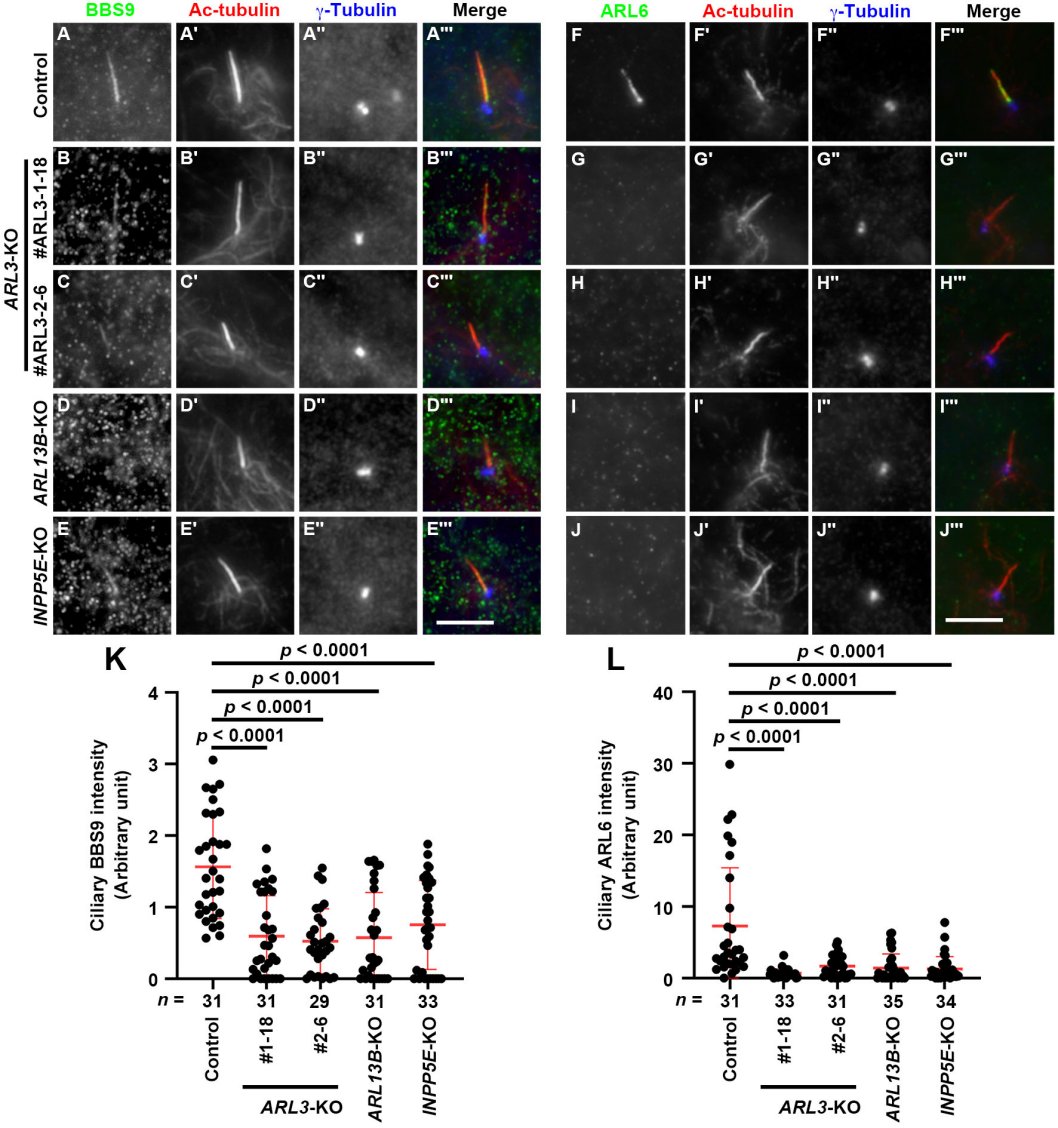
**Absence of the BBSome within cilia of *ARL3*-KO, *ARL13B*-KO, and *INPP5E*-KO cells.** Control RPE1 cells (A, F), the *ARL3*-KO cell lines #ARL3-1-18 (B, G) and #ARL3-2-6 (C, H), the *ARL13B*-KO cell line #ARL13B-1-2 (D, I), and the *INPP5E*-KO cell line #INPP5E-2-2 (E, J) were serum-starved for 24 h, and triply immunostained for either BBS9 (A–E) or ARL6 (F–J), Ac-tubulin (A′–J′), and γ-tubulin (A″–J″). Scale bars, 5 µm. (K, L) Relative ciliary staining intensities of BBS9 (K) and ARL6 (L) in control, *ARL3*-KO, *ARL13B*-KO, and *INPP5E*-KO cells were estimated and expressed as scatter plots. Horizontal lines indicate the means, and error bars are the SDs. Statistical significances were calculated using one-way ANOVA followed by the Dunnett's multiple comparison test.

As described above, in *ARL3*-KO cells, export of GPR161 from cilia upon Hh pathway stimulation was significantly suppressed (Fig. 4), even though TULP3 and the IFT machinery were not substantially trapped within cilia, compared with those in *ARL13B*-KO and *INPP5E*-KO cells (Fig. 3). Although intraciliary retrograde protein trafficking requires the IFT machinery and the aid of the dynein-2 motor, exit of membrane proteins across the ciliary gate also requires the BBSome complexed with the IFT machinery (Lechtreck et al., 2013; Liew et al., 2014; Liu and Lechtreck, 2018; Nozaki et al., 2018; 2019; Ye et al., 2018). Therefore, we next analyzed the localization of the BBSome components in *ARL3*-KO, *ARL13B*-KO, and *INPP5E*-KO cells. In agreement with previous studies (Nozaki et al., 2018; Seo et al., 2011), immunostaining for BBS9 was found in cilia of control RPE1 cells, although at a relatively low level (Fig. 5A; also see Fig. 5K). In marked contrast, BBS9 signals were significantly decreased not only in *ARL3*-KO cells but also in *ARL13B*-KO and *INPP5E*-KO cells (Fig. 5B–E; also see Fig. 5K). Essentially the same results were obtained for ARL6; ARL6 was detected in cilia of control cells, and the ARL6 level was significantly decreased in the cilia of *ARL3*-KO, *ARL13B*-KO, and *INPP5E*-KO cells (Fig. 5F–J; also see Fig. 5L). These observations are in line with previous studies showing that exit of GPR161 from cilia is dependent on the BBSome (Nozaki et al., 2019; Ye et al., 2018).

### Direct binding of ciliary ARL13B to INPP5E determines its ciliary membrane localization

As observed above, some of the phenotypes of *ARL3*-KO and *ARL13B*-KO cells are similar to each other, in particular, the delocalization of INPP5E from cilia, whereas other phenotypes are different. These observations are not necessarily compatible with the notion that ARL13B activates ARL3 as its GEF, and that activated ARL3 in turn determines the ciliary membrane targeting of INPP5E by stimulating its release from PDE6D. In addition, we recently showed that INPP5E requires ARL13B for its ciliary membrane retention but does not necessarily require ARL13B for its entry into cilia across the ciliary gate (Qiu et al., 2021). We therefore aimed to clarify whether the ciliary membrane localization of INPP5E correlates with its binding to ARL13B.

For this purpose, we focused on the data from a sophisticated biochemical study on ARL13B as an ARL3-GEF, in which the T35N mutant and three JBTS mutants (R79Q, Y86C, and R200C) of ARL13B were shown to lack ARL3-GEF activity (Ivanova et al., 2017). In this context, it is important to note that we previously showed that ARL13B(T35N) had reduced ability to bind INPP5E, and partially restored the ciliary membrane localization of INPP5E when expressed in *ARL13B*-KO cells (Nozaki et al., 2017).

**Fig. 6.**
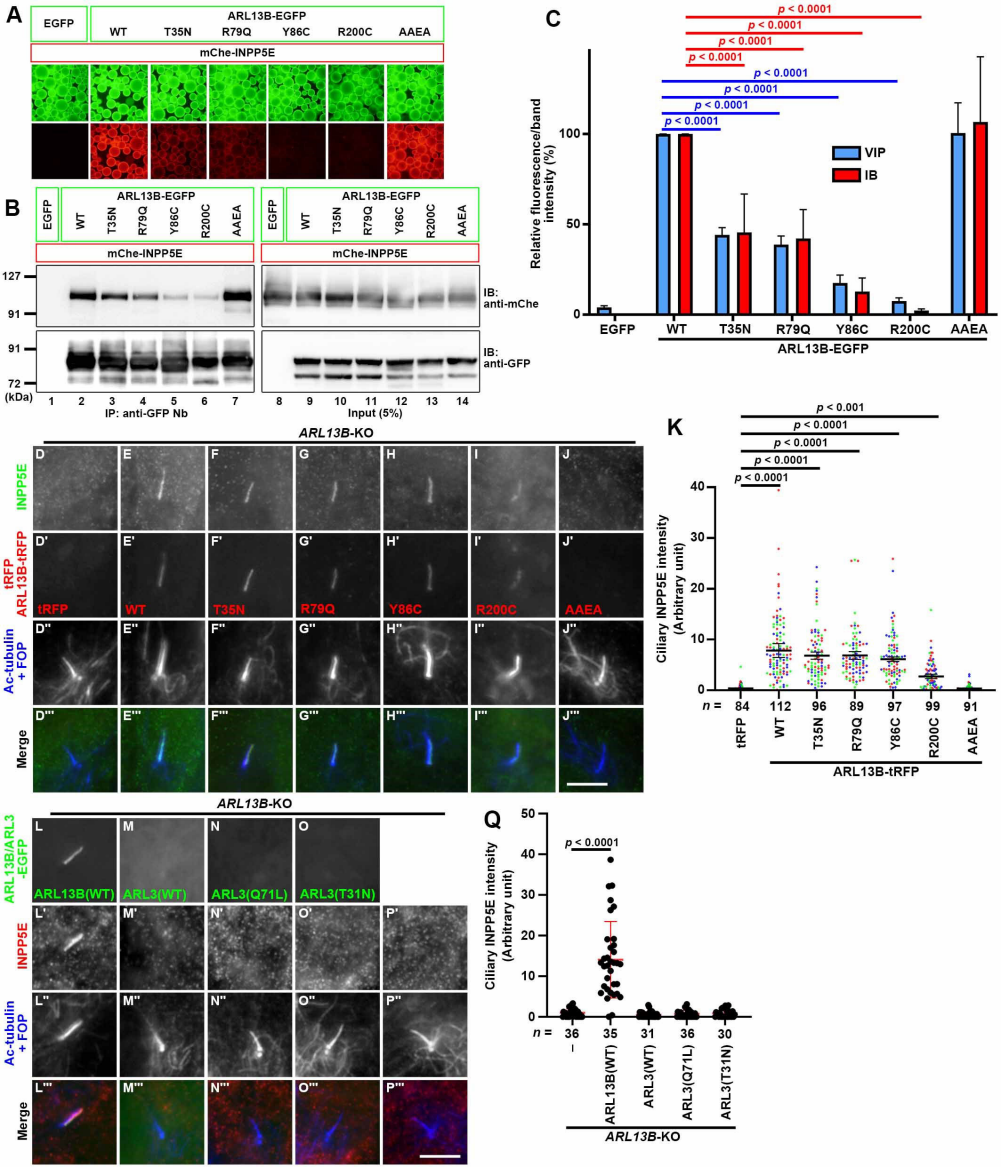
**Ciliary localization of INPP5E correlates with its binding to ARL13B.** (A, B) Lysates of cells coexpressing an EGFP-fused ARL13B construct as indicated and mChe-INPP5E were subjected to the VIP assay using GST–anti-GFP Nb (A), followed by immunoblotting analysis using anti-mChe and anti-GFP antibodies (B). (C) The fluorescence intensities of (A) and the band intensities (B) of mChe-INPP5E were measured, and the relative intensities are represented as bar graphs with the intensity in HEK293T cells expressing ARL13B(WT)-EGFP set as 100%. Values are shown as the means±SD of three independent experiments. Statistical significances among multiple cell lines were calculated using one-way ANOVA followed by the Dunnett's multiple comparison test. (D–J) *ARL13B*-KO cells (#ARL13B-1-2) expressing the tRFP-fused ARL13B constructs indicated were serum-starved for 24 h and immunostained for INPP5E (D–J) and Ac-tubulin+FOP (D″–J″). (K) The relative ciliary staining intensities of INPP5E in *ARL13B*-KO cells stably expressing tRFP (D) or the tRFP-fused ARL13B constructs indicated (E–J) are represented as scatter plots. Different colored dots represent three independent experiments, horizontal lines indicate the means, and error bars are the SDs. Statistical significances among multiple cell lines were calculated using one-way ANOVA followed by the Dunnett's multiple comparison test. (L–P) *ARL13B*-KO cells (#ARL13B-1-2), which stably express EGFP-fused ARL13B(WT) (L), ARL3(WT) (M), ARL3(Q71L) (N), or ARL3(T31N) (O) were serum starved for 24 h and triply immunostained for GFP, INPP5E, and Ac-tubulin+FOP. (Q) Relative ciliary staining intensities of INPP5E in individual cells shown in (L′)–(P′) were estimated and expressed as scatter plots. The horizontal lines indicate the means, and the error bars indicate the SDs. Statistical significances were calculated using one-way ANOVA followed by the Dunnett's multiple comparison test.

We therefore analyzed whether these JBTS mutants could bind INPP5E and restore the ciliary membrane localization of INPP5E in *ARL13B*-KO cells. As shown in Fig. 6A and B, the VIP assay and subsequent immunoblotting analysis showed that ARL13B(T35N), ARL13B(R79Q), ARL13B(Y86C), and ARL13B(R200C) had reduced ability to bind INPP5E, in comparison with ARL13B(WT) (also see Fig. 6C). Among these ARL13B mutants, ARL13B(Y86C) and ARL13B(R200C) had much lower abilities to bind INPP5E than ARL13B(WT). In contrast, the ARL13B(AAEA) mutant, in which the ciliary localization sequence RVEP (Higginbotham et al., 2012; Mariani et al., 2016) is mutated to AAEA, retained the ability to bind INPP5E, as described previously (Nozaki et al., 2017).

We then expressed these ARL13B mutants in *ARL13B*-KO cells to analyze whether they could restore the normal ciliary membrane localization of INPP5E. As shown in Fig. 6E (also see Fig. 6K), the expression of tRFP-fused ARL13B(WT) in *ARL13B*-KO cells restored the ciliary membrane localization of INPP5E. As reported previously (Nozaki et al., 2017), the expression of ARL13B(T35N)-tRFP significantly restored the INPP5E localization within cilia (Fig. 6F; also see Fig. 6K). The expression of ARL13B(R79Q)-tRFP and ARL13B(Y86C)-tRFP in *ARL13B*-KO cells also restored ciliary INPP5E localization (Fig. 6G, H; also see Fig. 6K). ARL13B(R200C)-tRFP also rescued the INPP5E delocalization to some extent (Fig. 6I; also see Fig. 6K). Thus, the ability of the ARL13B mutants to restore ciliary INPP5E localization in *ARL13B*-KO cells was independent of their ability as an ARL3-GEF, but roughly correlated with their ability to interact with INPP5E (Fig. 6A and B). By contrast, ARL13B(AAEA)-tRFP, which was absent from cilia, did not restore ciliary INPP5E localization (Fig. 6J; also see Fig. 6K), confirming that the ciliary localization of ARL13B is essential for the ciliary targeting of INPP5E. These observations altogether demonstrate that INPP5E is retained on the ciliary membrane via its direct interaction with ciliary ARL13B.

In view of a previous biochemical study showing that not only ARL13B(T35N) but also the JBTS mutants lack ARL3-GEF activity (Ivanova et al., 2017), the above observations do not support the idea that the ARL3-GEF activity of ARL13B contributes to ciliary INPP5E localization. To support the notion that ARL13B is not involved in the targeting of INPP5E to cilia via activation of ARL3, we then analyzed whether the ciliary localization of INPP5E in *ARL13B*-KO cells could be restored by the exogenous expression of ARL3(Q71L), which is a GTPase-restricted (GTP-locked) mutant. As shown in Fig. 6M–O, the stable expression of either ARL3(WT), ARL3(Q71L), or ARL3(T31N) in *ARL13B*-KO cells did not restore the ciliary localization of INPP5E, although the expression of ARL13B(WT) restored INPP5E localization (Fig. 6L; also see Fig. 6Q). By contrast, as described above, when expressed in *ARL3*-KO cells as control experiments, ARL3(WT) and ARL3(Q71L), but not ARL3(T31N), significantly restored ciliary INPP5E localization, although neither of them was detectable within cilia (Fig. 2J–N).

Taken altogether, our observations support the hypothesis that the direct interaction of INPP5E with ARL13B is crucial for the retention of INPP5E on the ciliary membrane, and hence it is unlikely that the role of ARL13B as an ARL3-GEF makes a major contribution to the ciliary localization of INPP5E.

## DISCUSSION

Two ARL GTPases, ARL3 and ARL13B, participate in the localization of INPP5E on the ciliary membrane. There are two possible roles of ARL13B in determining INPP5E ciliary localization, although these are not mutually exclusive (Fisher et al., 2020). One is the direct role, in which ARL13B localized on the ciliary membrane retains INPP5E via binding its CTS. The other is the indirect role, in which ARL13B activates ARL3, which in turn promotes the release of prenylated INPP5E from PDE6D to be anchored to the ciliary membrane. The data presented in this study, together with those presented in our previous study, support the direct role of ARL13B at least within cilia, independently of its ARL3-GEF activity, for the following reasons: (i) the interaction of INPP5E with ARL13B via its CTS is required for its retention on the ciliary membrane but not for its entry into cilia (Qiu et al., 2021); (ii) the localization of ARL13B on the ciliary membrane is required for the ciliary localization of INPP5E (Fig. 6E, J, K); (iii) analyses using ARL13B mutants, which were demonstrated to lack ARL3-GEF activity (Ivanova et al., 2017), indicate that the ciliary localization of INPP5E does not correlate with ARL3-GEF activity of the ARL13B mutants but roughly correlates with their direct binding to INPP5E (Fig. 6E–I, K); and (iv) the GTP-locked mutant, ARL3(Q71L), did not restore the ciliary localization of INPP5E when expressed in *ARL13B*-KO cells (Fig. 6N, Q), although it did restore the localization of INPP5E in *ARL3*-KO cells (Fig. 2K, N).

Our data are compatible with a model in which ARL3 and ARL13B are involved in distinct steps of the ciliary targeting of INPP5E. Namely, ARL3 promotes the release of INPP5E from PDE6D, after which ARL13B binds INPP5E to retain it on the ciliary membrane, as ARL13B cannot interact with INPP5E when it is complexed with PDE6D (Fig. 1J, K). This model is associated with the crucial issue regarding the timing of release of INPP5E from PDE6D and its subsequent anchorage to the membrane via its prenyl moiety; namely, before or after its passage across the ciliary gate. If ARL3 can be activated on the cytoplasmic side of the ciliary gate, INPP5E is expected to undergo ARL3-mediated release from PDE6D on the cytoplasmic side, and become anchored to the plasma membrane via its prenyl moiety, after which it is expected to pass the ciliary gate by lateral diffusion. In *ARL13B*-KO cells, INPP5E can be anchored to the membrane and enter cilia by lateral diffusion, but fails to be retained on the ciliary membrane owing to the lack of ciliary ARL13B (Qiu et al., 2021). In this context, it is interesting to note that mutations in genes encoding various TZ proteins, such as TMEM216/MKS2/JBTS2 and TMEM67/MKS3/JBTS6, are known to cause JBTS (Braun and Hildebrandt, 2017; Parisi and Glass, 2017); therefore, impaired integrity of the TZ might result in its impaired role as a diffusion barrier (Okazaki et al., 2020). If INPP5E in complex with PDE6D can permeate the ciliary gate into cilia, activated ARL3 within cilia is expected to allosterically interact with PDE6D to release INPP5E, which is in turn recognized by ARL13B. Although we were unable to detect the ciliary localization of ARL3 (see Fig. 2J–L), ARL3 is expected to freely permeate the ciliary gate owing to its relatively small size (Lin et al., 2013; Takao and Verhey, 2016) if it exists as a soluble protein, because ARL3 was suggested not to undergo N-myristoylation unlike typical ARF GTPases (Fansa and Wittinghofer, 2016). In *ARL3*-KO cells, even if INPP5E in complex with PDE6D is able to permeate the ciliary gate into cilia, it is not retained on the ciliary membrane, as ARL13B cannot interact with INPP5E in complex with PDE6D, which must be released by ARL3 (Fig. 1). As the ARL3-GEF activity of ARL13B is dispensable for ciliary INPP5E localization, how ARL3 is activated and whether ARL3 is activated outside or inside of cilia are important issues to be addressed in the future.

Not only in *ARL13B*-KO and *INPP5E*-KO cells but also in *ARL3*-KO cells, the stable localization of INPP5E on the ciliary membrane was abolished and export of GPR161 from cilia upon SAG stimulation was suppressed (Figs. 2 and 4). As the TULP3 adaptor, which connects ciliary GPCR with the IFT machinery (Badgandi et al., 2017; Mukhopadhyay et al., 2010) and mediates GPCR entry into cilia across the ciliary gate (Badgandi et al., 2017) binds to PtdIns(4,5)P_2_, but not to PtdIns(4)P (Mukhopadhyay et al., 2010), previous studies analyzing cells derived from *Inpp5e*-KO mice concluded that PtdIns(4,5)P_2_ buildup in INPP5E-deficient cilia causes an increase in the ciliary GPR161 level owing to the accumulation of the IFT machinery together with TULP3, which binds PtdIns(4,5)P_2_ (Chávez et al., 2015; Garcia-Gonzalo et al., 2015; Nakatsu, 2015). However, our data on the localization of IFT proteins and TULP3 in *ARL3*-KO cells appear to be incompatible with the notion proposed in the previous studies; namely, the IFT-B and IFT-A proteins and TULP3 were not enriched in *ARL3*-KO cilia, whereas they were significantly enriched in *ARL13B*-KO and *INPP5E*-KO cilia (Fig. 3). Therefore, whether the INPP5E–PDE6D complex can modulate the ciliary phosphoinositide composition and whether there is a correlation between the ciliary TULP3 level and the phosphoinositide composition on the ciliary membrane are important issues to be addressed, although our attempts to investigate changes in the levels of ciliary PtdIns(4)P and PtdIns(4,5)P_2_ have so far been unsuccessful for technical reasons.

On the other hand, the ciliary levels of the BBSome and the ARL6/BBS3 GTPase were consistently decreased in *ARL3*-KO, *ARL13B*-KO, and *INPP5E*-KO cells (Fig. 5). As exit of ciliary GPCRs across the ciliary gate, in particular, the exit of GPR161 upon stimulation of the Hh pathway, requires the BBSome complexed with the IFT machinery (Liew et al., 2014; Nozaki et al., 2018; 2019; Ye et al., 2018), decreased BBSome levels (Fig. 5) and GPR161 accumulation within cilia (Fig. 4) are correlated with each other in these KO cells. However, for unknown reasons, the exogenous expression of ARL3 was unable to restore the localization of the BBSome within the cilia of *ARL3*-KO cells. The observations in *ARL3*-KO cells were similar to those in *BBS1*-KO cells, as previously reported; unexpectedly, the exogenous expression of BBS1 in *BBS1*-KO cells did not restore the localization of BBS9 or ARL6 within cilia, whereas the SAG-stimulated exit of GPR161 was restored (Nozaki et al., 2018). It will therefore be necessary in the future to investigate the reason why the BBSome is delocalized in *ARL3*-KO, *ARL13B*-KO, and *INPP5E*-KO cells, and whether the impaired exit of GPR161 correlates with delocalization of the BBSome in these KO cells. The molecular and cellular mechanisms underlying how mutations in INPP5E, ARL13B, PDE6D, and ARL3 result in the common phenotypes of JBTS are also important issues to be addressed in the future.

## MATERIALS AND METHODS

### Plasmids, antibodies, reagents, and cell lines

cDNAs for human ARL3 (NM_004311) and PDE6D (NM_002601) were obtained from a cDNA library by PCR amplification. Expression vectors used in this study are listed in Table S1; some of them were constructed in our previous studies (Nozaki et al., 2017; Qiu et al., 2021). The antibodies used in this study are listed in Table S2. GST-tagged anti-GFP Nb prebound to glutathione-Sepharose 4B beads were prepared as described previously (Katoh et al., 2015; 2018). Polyethylenimine Max and SAG were purchased from Polysciences and Enzo Life Sciences, respectively. HEK293T cells and hTERT-RPE1 cells were obtained from RIKEN BioResource Research Center (RBC2202) and American Type Culture Collection (CRL-4000), respectively.

### VIP assay and immunoblotting analysis

The VIP assay and subsequent immunoblotting analyses were carried out as described previously (Katoh et al., 2015; 2018) with slight modifications (Nishijima et al., 2017), as follows: HEK293T cells expressing EGFP-tagged, tRFP-tagged, and tBFP-tagged proteins were lysed in lysis buffer (50 mM HEPES, pH 7.4, 100 mM KCl, 1 mM DTT, 5 mM NaCl, 3 mM MgCl_2_, 0.5% v/v Triton X100, and 10% w/v glycerol). The fluorescence on the beads was observed using an all-in-one type fluorescence microscope (Biozero BZ-8000, Keyence) or a confocal laser-scanning microscope (A1R-MP, Nikon) as described previously (Katoh et al., 2015).

### Establishment of KO cell lines using the CRISPR/Cas9 system

The targeted disruption of genes in hTERT-RPE1 cells by CRISPR/Cas9-mediated homology-independent DNA repair was performed as previously described (Katoh et al., 2017), with minor modifications (Nakamura et al., 2020; Tsurumi et al., 2019). Briefly, single-guide RNA (sgRNA) sequences targeting the human *ARL3* gene (see Table S3) were designed using CRISPOR (Haeussler et al., 2016). Double-stranded oligonucleotides for the target sequences were inserted into an all-in-one sgRNA expression vector, pHiFiCas9-2×sgRNA (Addgene 162277), in which the eSpCAS9 sequence in peSpCAS9(1.1)-2×sgRNA (Katoh et al., 2017) was replaced with the high fidelity Cas9 sequence, HiFiCas9 (Vakulskas et al., 2018). hTERT-RPE1 cells cultured on a 12-well plate to approximately 3.0×10^5^ cells were transfected with 1 µg of the all-in-one vector and 0.25 µg of the donor knock-in vector, pDonor-tBFP-NLS-Neo(universal) (Addgene 80767), using X-tremeGENE9 Reagent (Roche Applied Science). After culturing in the presence of G418 (600 µg/mL), cells expressing nuclear tBFP were sorted using the SH800S cell sorter (SONY) at the Medical Research Support Center of Kyoto University. To confirm the disruption of the *ARL3* gene in the isolated cell lines, genomic DNA extracted from the isolated cells was subjected to PCR using GoTaq^®^ Master Mixes (Promega) and three sets of primers (Table S3), to distinguish the following three states of integration of the donor knock-in vector: forward integration (Fig. S1A, b and b′), reverse integration (c and c′), and no integration with a small indel (a and a′). Direct sequencing of the genomic PCR products was performed to confirm the disruption of both alleles of the *ARL3* gene. The *ARL13B*-KO cell line (#ARL13B-1-2) and *INPP5E*-KO cell line (#INPP5E-2-2) were established in our previous studies (Nozaki et al., 2017; Qiu et al., 2021).

### Preparation of lentiviral vectors and cells stably expressing ARL3 and ARL13B constructs

Lentiviral vectors for the stable expression of ARL3 and ARL13B constructs were prepared as previously described (Nozaki et al., 2017; Takahashi et al., 2012). Briefly, the ARL3-EGFP and ARL13B-tRFP constructs in pRRLsinPPT were transfected into HEK293T cells using Polyethylenimine Max together with the packaging plasmids (pRSV-REV, pMD2.g, and pMDLg/pRRE; kindly provided by Peter McPherson, McGill University) (Thomas et al., 2009). The culture medium was replaced 8 h after transfection, and collected at 24, 36, and 48 h after transfection. The viral particle-containing medium was passed through a 0.45-µm filter and centrifuged at 32,000×***g*** at 4°C for 4 h to precipitate viral particles, which were resuspended in Opti-MEM (Invitrogen) and stored at −80°C until use. *ARL3*-KO and *ARL13B*-KO cells stably expressing an ARL3 or ARL13B construct were prepared by addition of the lentiviral suspension to the culture medium.

### Immunofluorescence analysis

Induction of ciliogenesis and subsequent immunofluorescence analysis of hTERT-RPE1 cells were performed as described previously (Nozaki et al., 2017; Takahashi et al., 2012). The immunostained cells were observed using an Axiovert 200M or Axio Observer microscope (Carl Zeiss). For quantification analysis, all images were acquired under the same setting and imported as TIFF files using ImageJ software. A ROI was constructed by drawing a line of three-point width along the ciliary signal of Ac-tubulin using a segmented line tool. To correct for local background intensity, the ROI was duplicated and dragged to a nearby region. Statistical analyses were performed using GraphPad Prism8 (Version 8.4.3; GraphPad Software, Inc.).

## Supplementary Material

Supplementary information

## References

[BIO058843C1] Alkanderi, S., Molinari, E., Shaheen, R., Elmaghloob, Y., Stephen, L. A., Sammut, V., Ramsbottom, S. A., Srivastava, S., Cairns, G., Edwards, N. et al. (2018). *ARL3* mutations cause Joubert syndrome by disrupting ciliary protein composition. *Am. J. Hum. Genet.* 103, 612-620. 10.1016/j.ajhg.2018.08.015 30269812 PMC6174286

[BIO058843C2] Badgandi, H. B., Hwang, S., Shimada, I. S., Loriot, E. and Mukhopadhyay, S. (2017). Tubby family proteins are adaptors for ciliary trafficking of integral membrane proteins. *J. Cell Biol.* 216, 743-760. 10.1083/jcb.201607095 28154160 PMC5350516

[BIO058843C3] Bangs, F. and Anderson, K. V. (2017). Primary cilia and mammalian hedgehog signaling. *Cold Spring Harb. Perspect. Biol.* 9, a028175. 10.1101/cshperspect.a028175 27881449 PMC5411695

[BIO058843C4] Bielas, S. L., Silhavy, J. L., Brancati, F., Kisseleva, M. V., Al-Gazali, L., Sztriha, L., Bayoumi, R. A., Zaki, M. S., Abdel-Aleem, A., Rosti, R. O. et al. (2009). Mutations in *INPP5E*, encoding inositol polyphosphate-5-phosphatase E, link phosphatidyl inositol signaling to the ciliopathies. *Nat. Genet.* 41, 1032-1036. 10.1038/ng.423 19668216 PMC2746682

[BIO058843C5] Braun, D. A. and Hildebrandt, F. (2017). Ciliopathies. *Cold Spring Harb. Perspect. Biol.* 9, a028191. 10.1101/cshperspect.a028191 27793968 PMC5334254

[BIO058843C6] Cantagrel, V., Silhavy, J. L., Bielas, S. L., Swistun, D., Marsh, S. E., Bertrand, J. Y., Audollent, S., Attié-Bitach, T., Holden, K. R., Dobyns, W. B. et al. (2008). Mutations in the cilia gene *ARL13B* lead to the classical form of Joubert syndrome. *Am. J. Hum. Genet.* 83, 170-179. 10.1016/j.ajhg.2008.06.023 18674751 PMC2495072

[BIO058843C7] Chávez, M., Ena, S., Van Sande, J., de Kerchove d'Exaerde, A., Schurmans, S. and Schiffmann, S. N. (2015). Modulation of ciliary phosphoinositide content regulates trafficking and sonic hedgehog signaling output. *Dev. Cell* 34, 338-350. 10.1016/j.devcel.2015.06.016 26190144

[BIO058843C8] Conduit, S. E. and Vanhaesebroeck, B. (2020). Phosphoinositide lipids in primary cilia biology. *Biochem. J.* 477, 3541-3565. 10.1042/BCJ20200277 32970140 PMC7518857

[BIO058843C9] Dateyama, I., Sugihara, Y., Chiba, S., Ota, R., Nakagawa, R., Kobayashi, T. and Itoh, H. (2019). RABL2 positively controls localization of GPCRs in mammalian primary cilia. *J. Cell Sci.* 132, jcs224428. 10.1242/jcs.224428 30578315

[BIO058843C10] Eguether, T., San Agustin, J. T., Keady, B. T., Jonassen, J. A., Liang, Y., Francis, R., Tobita, K., Johnson, C. A., Abdelhamed, Z. A., Lo, C. W. et al. (2014). IFT27 links the BBSome to IFT for maintenance of the ciliary signaling compartment. *Dev. Cell* 31, 279-290. 10.1016/j.devcel.2014.09.011 25446516 PMC4254547

[BIO058843C11] El Maghloob, Y., Sot, B., Mcllwraith, M. J., Garcia, E., Yelland, T. and Ismail, S. (2021). ARL3 activation requires the co-GEF BART and effector-mediated turnover. *eLife* 10, e64624. 10.7554/eLife.64624 33438581 PMC7817177

[BIO058843C12] Fansa, E. K. and Wittinghofer, A. (2016). Sorting of lipidated cargo by the Arl2/Arl3 system. *Small GTPases* 7, 222-230. 10.1080/21541248.2016.1224454 27806215 PMC5129900

[BIO058843C13] Fansa, E. K., Kösling, S. K., Zent, E., Wittinghofer, A. and Ismail, S. (2016). PDE6δ-mediated sorting of INPP5E into the cilium is determined by cargo-carrier affinity. *Nat. Commun.* 7, 1-19. 10.1038/ncomms11366 PMC5512577 27063844

[BIO058843C14] Fisher, S., Kuna, D., Caspary, T., Kahn, R. A. and Sztul, E. (2020). ARF family GTPases with links to cilia. *Am. J. Physiol. Cell Physiol.* 319, C404-C418. 10.1152/ajpcell.00188.2020 32520609 PMC7500214

[BIO058843C15] Garcia-Gonzalo, F. R. and Reiter, J. F. (2017). Open sesame: how transition fibers and the transition zone control ciliary composition. *Cold Spring Harb. Perspect. Biol.* 9, a028134. 10.1101/cshperspect.a028134 27770015 PMC5287074

[BIO058843C16] Garcia-Gonzalo, F. R., Phua, S. C., Roberson, E. C., Garcia, G., III, Abedin, M., Schurmans, S., Inoue, T. and Reiter, J. F. (2015). Phosphoinositides regulate ciliary protein trafficking to modulate Hedgehog signaling. *Dev. Cell* 34, 400-409. 10.1016/j.devcel.2015.08.001 26305592 PMC4557815

[BIO058843C17] Gigante, E. D. and Caspary, T. (2020). Signaling in the primary cilum through the lens of the Hedgehog pathway. *Wiley Interdiscip. Rev. Dev. Biol.* 9, e377. 10.1002/wdev.377 32084300 PMC7444278

[BIO058843C18] Gonçalves, J. and Pelletier, L. (2017). The ciliary transition zone: finding the pieces and assembling the gate. *Mol. Cells* 40, 243-253. 10.14348/molcells.2017.0054 28401750 PMC5424270

[BIO058843C19] Gotthardt, K., Lokaj, M., Koerner, C., Falk, N., Gießl, A. and Wittinghofer, A. (2015). A G-protein activation cascade from Arl13B to Arl3 and implications for ciliary targeting of lipidated proteins. *eLife* 4, e11859. 10.7554/eLife.11859 26551564 PMC4868535

[BIO058843C20] Haeussler, M., Schönig, K., Eckert, H., Eschstruth, A., Mianné, J., Renaud, J.-B., Schneider-Maunoury, S., Shkumatava, A., Teboul, L., Kent, J. et al. (2016). Evaluation of off-target and on-target scoring algorithms and integration into the guide RNA selection tool CRISPOR. *Genome Biol.* 17, 148. 10.1186/s13059-016-1012-2 27380939 PMC4934014

[BIO058843C21] Han, S., Miyoshi, K., Shikada, S., Amano, G., Wang, Y., Yoshimura, T. and Katayama, T. (2019). TULP3 is required for localization of membrane-associated proteins ARL13B and INPP5E to primary cilia. *Biochem. Biophys. Res. Commun.* 509, 227-234. 10.1016/j.bbrc.2018.12.109 30583862

[BIO058843C22] Hanke-Gogokhia, C., Wu, Z., Gerstner, C. D., Frederick, J. M., Zhang, H. and Baehr, W. (2016). Arf-like protein 3 (ARL3) regulates protein trafficking and ciliogenesis in mouse photoreceptors. *J. Biol. Chem.* 290, 7142-7155. 10.1074/jbc.M115.710954 PMC4807295 26814127

[BIO058843C23] Higginbotham, H., Eom, T.-Y., Mariani, L. E., Bachleda, A., Hirt, J., Gukassyan, V., Cusack, C. L., Lai, C., Caspary, T. and Anton, E. S. (2012). Arl13b in primary cilia regulates the migration and placement of interneurons in the developing cerebral cortex. *Dev. Cell* 23, 925-938. 10.1016/j.devcel.2012.09.019 23153492 PMC3529475

[BIO058843C24] Hirano, T., Katoh, Y. and Nakayama, K. (2017). Intraflagellar transport-A complex mediates ciliary entry as well as retrograde trafficking of ciliary G protein-coupled receptors. *Mol. Biol. Cell* 28, 429-439. 10.1091/mbc.e16-11-0813 27932497 PMC5341726

[BIO058843C25] Humbert, M. C., Weihbrecht, K., Searby, C. C., Li, Y., Pope, R. M., Sheffield, V. C. and Seo, S. (2012). ARL13B, PDE6D, and CEP164 form a functional network for INPP5E ciliary targeting. *Proc. Natl. Acad. Sci. USA* 109, 19691-19696. 10.1073/pnas.1210916109 23150559 PMC3511769

[BIO058843C26] Ishida, Y., Kobayashi, T., Chiba, S., Katoh, Y. and Nakayama, K. (2021). Molecular basis of ciliary defects caused by compound heterozygous *IFT144/WDR19* mutations found in cranioectodermal dysplasia. *Hum. Mol. Genet.* 30, 213-225. 10.1093/hmg/ddab034 33517396

[BIO058843C27] Ishikawa, H. and Marshall, W. F. (2011). Ciliogenesis: building the cell's antenna. *Nat. Rev. Mol. Cell Biol.* 12, 222-234. 10.1038/nrm3085 21427764

[BIO058843C28] Ishikawa, H., Thompson, J., Yates, J. R., III and Marshall, W. F. (2012). Proteomic analysis of mammalian primary cilia. *Curr. Biol.* 22, 414-419. 10.1016/j.cub.2012.01.031 22326026 PMC3298568

[BIO058843C29] Ismail, S. A., Chen, Y.-X., Rusinova, A., Chandra, A., Bierbaum, M., Gremer, L., Triola, G., Waldmann, H., Bastiaens, P. I. and Wittinghofer, A. (2011). Arl2-GTP and Arl3-GTP regulates a GDI-like transport system for farnesylated cargo. *Nat. Chem. Biol.* 7, 942-949. 10.1038/nchembio.686 22002721

[BIO058843C30] Ivanova, A. A., Caspary, T., Syfriend, N. T., Duong, D. M., West, A. B., Liu, Z. and Kahn, R. A. (2017). Biochemical characterization of purified mammalian ARL13B protein indicates that it is an atypical GTPase and ARL3 guanine nucleotide exchange factor (GEF). *J. Biol. Chem.* 292, 11091-11108. 10.1074/jbc.M117.784025 28487361 PMC5491791

[BIO058843C31] Jacoby, M., Cox, J. J., Gayral, S., Hampshire, D. J., Ayub, M., Blockmans, M., Pernot, E., Kisseleva, M. V., Compère, P., Schiffmann, S. N. et al. (2009). *INPP5E* mutations cause primary cilium signaling defects, ciliary instability and ciliopathies in human and mouse. *Nat. Genet.* 41, 1027-1031. 10.1038/ng.427 19668215

[BIO058843C32] Jensen, V. L. and Leroux, M. R. (2017). Gates for solubule and membrane proteins, and two trafficking systems (IFT and LIFT), establish a dynamic ciliary signaling compartment. *Curr. Opin. Cell Biol.* 47, 83-91. 10.1016/j.ceb.2017.03.012 28432921

[BIO058843C33] Kanie, T., Abbott, K. L., Mooney, N. A., Plowey, E. D., Demeter, J. and Jackson, P. K. (2017). The CEP19-RABL2 GTPase complex binds to IFT-B to initiate intraflagellar transport at the ciliary base. *Dev. Cell* 42, 22-36. 10.1016/j.devcel.2017.05.016 28625565 PMC5556974

[BIO058843C34] Katoh, Y., Nozaki, S., Hartanto, D., Miyano, R. and Nakayama, K. (2015). Architectures of multisubunit complexes revealed by a visible immunoprecipitation assay using fluorescent fusion proteins. *J. Cell Sci.* 128, 2351-2362. 10.1242/jcs.168740 25964651

[BIO058843C35] Katoh, Y., Terada, M., Nishijima, Y., Takei, R., Nozaki, S., Hamada, H. and Nakayama, K. (2016). Overall architecture of the intraflagellar transport (IFT)-B complex containing Cluap1/IFT38 as an essential component of the IFT-B peripheral subcomplex. *J. Biol. Chem.* 291, 10962-10975. 10.1074/jbc.M116.713883 26980730 PMC4900248

[BIO058843C36] Katoh, Y., Michisaka, S., Nozaki, S., Funabashi, T., Hirano, T., Takei, R. and Nakayama, K. (2017). Practical method for targeted disruption of cilia-related genes by using CRISPR/Cas9-mediated homology-independent knock-in system. *Mol. Biol. Cell* 28, 898-906. 10.1091/mbc.e17-01-0051 28179459 PMC5385939

[BIO058843C37] Katoh, Y., Nakamura, K. and Nakayama, K. (2018). Visible immunoprecipitation (VIP) assay: a simple and versatile method for visual detection of protein-protein interactions. *Bio-protocol* 8, e2687. 10.21769/BioProtoc.2687 34179269 PMC8203940

[BIO058843C38] Katoh, Y., Chiba, S. and Nakayama, K. (2020). Practical method for superresolution imaging of primary cilia and centrioles by expansion microscopy using an amplibody for fluorescence signal amplification. *Mol. Biol. Cell* 31, 2195-2206. 10.1091/mbc.E20-04-0250 32726175 PMC7550703

[BIO058843C39] Kobayashi, T., Ishida, Y., Hirano, T., Katoh, Y. and Nakayama, K. (2021). Cooperation of the IFT-A complex with the IFT-B complex is required for ciliary retrograde protein trafficking and GPCR import. *Mol. Biol. Cell* 32, 45-56. 10.1091/mbc.E20-08-0556 33175651 PMC8098818

[BIO058843C40] Lechtreck, K.-F., Brown, J. M., Sampaio, J. L., Craft, J. M., Shevchenko, A., Evans, J. E. and Witman, G. B. (2013). Cycling of the signaling protein phospholipase D through cilia requires the BBSome only for the export phase. *J. Cell Biol.* 201, 249-261. 10.1083/jcb.201207139 23589493 PMC3628507

[BIO058843C41] Li, Y., Wei, Q., Zhang, Y., Ling, K. and Hu, J. (2010). The small GTPase ARL-13 and ARL-3 coordinate intraflagellar transport and ciliogenesis. *J. Cell Biol.* 189, 1039-1051. 10.1083/jcb.200912001 20530210 PMC2886347

[BIO058843C42] Liew, G. M., Ye, F., Nager, A. R., Murphy, J. P., Lee, J. S. H., Aguiar, M., Breslow, D. K., Gygi, S. P. and Nachury, M. V. (2014). The intraflagellar transport protein IFT27 promotes BBSome exit from cilia through the GTPase ARL6/BBS3. *Dev. Cell* 31, 265-278. 10.1016/j.devcel.2014.09.004 25443296 PMC4255629

[BIO058843C43] Lim, Y. S., Chua, C. E. L. and Tang, B. L. (2011). Rabs and other small GTPases in ciliary transport. *Biol. Cell* 103, 209-221. 10.1042/BC20100150 21488838

[BIO058843C44] Lin, Y.-C., Niewiadomski, P., Lin, B., Nakamura, H., Phua, S. C., Jiao, J., Levchenko, A., Inoue, T., Rohatgi, R. and Inoue, T. (2013). Chemically inducible diffusion trap at cilia reveals molecular sieve-like barrier. *Nat. Chem. Biol.* 9, 437-443. 10.1038/nchembio.1252 23666116 PMC3870470

[BIO058843C45] Liu, P. and Lechtreck, K. F. (2018). The Bardet-Biedl syndrome protein complex is an adaptor expanding the cargo of range of intraflagellar transport trains for ciliary export. *Proc. Natl. Acad. Sci. USA* 115, E934-E943. 10.1073/pnas.1713226115 29339469 PMC5798339

[BIO058843C46] Mariani, L. E., Bijlsma, M. F., Ivanova, A. I., Suciu, S. K., Kahn, R. A. and Caspary, T. (2016). Arl13b regulates Shh signaling from both inside and outside the cilium. *Mol. Biol. Cell* 27, 3780-3790. 10.1091/mbc.E16-03-0189 PMC5170560 27682584

[BIO058843C47] Mick, D. U., Rodrigues, R. B., Leib, R. D., Adams, C. M., Chien, A. S., Gygi, S. P. and Nachury, M. V. (2015). Proteomics of primary cilia by proximity labeling. *Dev. Cell* 35, 497-512. 10.1016/j.devcel.2015.10.015 26585297 PMC4662609

[BIO058843C48] Mukhopadhyay, S. and Rohatgi, R. (2014). G-protein-coupled receptors, Hedgehog signaling and primary cilia. *Sem. Cell Dev. Biol.* 33, 63-72. 10.1016/j.semcdb.2014.05.002 PMC4130902 24845016

[BIO058843C49] Mukhopadhyay, S., Wen, X., Chih, B., Nelson, C. D., Lane, W. S., Scales, S. J. and Jackson, P. K. (2010). TULP3 bridges the IFT-A complex and membrane phosphoinositides to promote trafficking of G protein-coupled receptors into primary cilia. *Genes Dev.* 24, 2180-2193. 10.1101/gad.1966210 20889716 PMC2947770

[BIO058843C50] Nachury, M. V. and Mick, D. U. (2019). Establishing and regulating the composition of cilia for signal transduction. *Nat. Rev. Mol. Cell Biol.* 20, 389-405. 10.1038/s41580-019-0116-4 30948801 PMC6738346

[BIO058843C51] Nakamura, K., Noguchi, T., Takahara, M., Omori, Y., Furukawa, T., Katoh, Y. and Nakayama, K. (2020). Anterograde trafficking of ciliary MAP kinase-like ICK/CILK1 by the intraflagellar transport machinery is required for intraciliary retrograde protein trafficking. *J. Biol. Chem.* 295, 13363-13376. 10.1074/jbc.RA120.014142 32732286 PMC7504932

[BIO058843C52] Nakatsu, F. (2015). A phosphoinositide code for primary cilia. *Dev. Cell* 34, 379-380. 10.1016/j.devcel.2015.08.008 26305588

[BIO058843C53] Nakayama, K. and Katoh, Y. (2018). Ciliary protein trafficking mediated by IFT and BBSome complexes with the aid of kinesin-2 and dynein-2 motors. *J. Biochem.* 163, 155-164. 10.1093/jb/mvx087 29272450

[BIO058843C54] Nakayama, K. and Katoh, Y. (2020). Architecture of the IFT ciliary trafficking machinery and interplay between its components. *Crit. Rev. Biochem. Mol. Biol.* 55, 179-196. 10.1080/10409238.2020.1768206 32456460

[BIO058843C55] Nishijima, Y., Hagiya, Y., Kubo, T., Takei, R., Katoh, Y. and Nakayama, K. (2017). RABL2 interacts with the intraflagellar transport B complex and CEP19 and participates in ciliary assembly. *Mol. Biol. Cell* 28, 1652-1666. 10.1091/mbc.E17-01-0017 28428259 PMC5469608

[BIO058843C56] Nozaki, S., Katoh, Y., Terada, M., Michisaka, S., Funabashi, T., Takahashi, S., Kontani, K. and Nakayama, K. (2017). Regulation of ciliary retrograde protein trafficking by the Joubert syndrome proteins ARL13B and INPP5E. *J. Cell Sci.* 130, 563-576. 10.1242/jcs.197004 27927754

[BIO058843C57] Nozaki, S., Katoh, Y., Kobayashi, T. and Nakayama, K. (2018). BBS1 is involved in retrograde trafficking of ciliary GPCRs in the context of the BBSome complex. *PLoS One* 13, e0195005. 10.1371/journal.pone.0195005 29590217 PMC5874067

[BIO058843C58] Nozaki, S., Castro Araya, R. F., Katoh, Y. and Nakayama, K. (2019). Requirement of IFT-B–BBSome complex interaction in export of GPR161 from cilia. *Biol. Open* 8, bio043786. 10.1242/bio.043786 31471295 PMC6777367

[BIO058843C59] Okazaki, M., Kobayashi, T., Chiba, S., Takei, R., Liang, L., Nakayama, K. and Katoh, Y. (2020). Formation of the B9-domain protein complex MKS1–B9D2–B9D1 is essential as a diffusion barrier for ciliary membrane proteins. *Mol. Biol. Cell* 31, 2259-2268. 10.1091/mbc.E20-03-0208 32726168 PMC7550698

[BIO058843C60] Parisi, M. and Glass, I. (2017). Joubert syndrome. In *GeneReviews^®^ [Internet]* (ed. M.P. Adam, H.H. Ardinger, R.A. Pagon, S.E. Wallace, L.J.H. Bean, K. Stephens and A. Amemiya). Seatle, WA: University of Washington, Bookshelf ID: NBK1325. 20301500

[BIO058843C61] Park, J., Lee, J., Shim, J., Han, W., Lee, J., Bae, Y. C., Chung, Y. D., Kim, C. H. and Moon, S. J. (2013). dTULP, the *Drosophila melanogaster* homolog of Tubby, regulates transient receptor potential channel localization in cilia. *PLoS Genet.* 9, e1003814. 10.1371/journal.pgen.1003814 24068974 PMC3778012

[BIO058843C62] Picariello, T., Brown, J. M., Hou, Y., Swank, G., Cochran, D. A., King, O. D., Lechtreck, K., Pazour, G. J. and Witman, G. B. (2019). A global analysis of IFT-A function reveals specialization for transport of membrane-associated proteins into cilia. *J. Cell Sci.* 132, jcs220749. 10.1242/jcs.220749 30659111 PMC6382014

[BIO058843C63] Qiu, H., Fujisawa, S., Nozaki, S., Katoh, Y. and Nakayama, K. (2021). Interaction of INPP5E with ARL13B is essential for its ciliary membrane retention but dispensable for its ciliary entry. *Biol. Open* 10, bio057653. 10.1242/bio.057653 33372066 PMC7860134

[BIO058843C64] Reiter, J. F. and Leroux, M. R. (2017). Genes and molecular pathways underpinning ciliopathies. *Nat. Rev. Mol. Cell Biol.* 18, 533-547. 10.1038/nrm.2017.60 28698599 PMC5851292

[BIO058843C65] Schrick, J. J., Vogel, P., Abuin, A., Hampton, B. and Rice, D. S. (2006). ADP-ribosylation factor-like 3 is involved in kidney and photoreceptor development. *Am. J. Pathol.* 168, 1288-1298. 10.2353/ajpath.2006.050941 16565502 PMC1606550

[BIO058843C66] Seo, S., Zhang, Q., Bugge, K., Breslow, D. K., Searby, C. C., Nachury, M. V. and Sheffield, V. C. (2011). A novel protein LZTFL1 regulates ciliary trafficking of the BBSome and Smoothened. *PLoS Genet.* 7, e1002358. 10.1371/journal.pgen.1002358 22072986 PMC3207910

[BIO058843C67] Shi, X., Garcia, G., III, Van De Weghe, J. C., McGorty, R., Pazour, G. J., Doherty, D., Huang, B. and Reiter, J. F. (2017). Super-resolution microscopy reveals that disruption of ciliary transition-zone architecture causes Joubert syndrome. *Nat. Cell Biol.* 19, 1178-1188. 10.1038/ncb3599 28846093 PMC5695680

[BIO058843C68] Stephen, L. A. and Ismail, S. (2016). Shuttling and sorting of lipid-modified cargo into the cilia. *Biochem. Soc. Trans.* 44, 1273-1280. 10.1042/BST20160122 27911709

[BIO058843C69] Takahashi, S., Kubo, K., Waguri, S., Yabashi, A., Shin, H.-W., Katoh, Y. and Nakayama, K. (2012). Rab11 regulates exocytosis of recycling vesicles at the plasma membrane. *J. Cell Sci.* 125, 4049-4057. 10.1242/jcs.102913 22685325

[BIO058843C70] Takao, D. and Verhey, K. J. (2016). Gated entry into the ciliary compartment. *Cell. Mol. Life Sci.* 73, 119-127. 10.1007/s00018-015-2058-0 26472341 PMC4959937

[BIO058843C71] Taschner, M. and Lorentzen, E. (2016). The intraflagellar transport machinery. *Cold Spring Harb. Perspect. Biol.* 8, a028092. 10.1101/cshperspect.a028092 27352625 PMC5046692

[BIO058843C72] Thomas, S., Ritter, B., Verbich, D., Sanson, C., Bourbonnière, L., McKinney, R. A. and McPherson, P. S. (2009). Intersectin regulates dendritic spine development and somatodendritic endocytosis but not synaptic vesicle recycling in hippocampal neurons. *J. Biol. Chem.* 284, 12410-12419. 10.1074/jbc.M809746200 19258322 PMC2673308

[BIO058843C73] Thomas, S., Wright, K. J., Le Corre, S., Micalizzi, A., Romani, M., Abhyankar, A., Saada, J., Perrault, I., Amiel, J., Litzler, J. et al. (2014). A homozygous PDE6D mutation in Joubert syndrome impairs targeting of farnesylated INPP5E protein to the primary cilium. *Hum. Mut.* 35, 137-146. 10.1002/humu.22470 24166846 PMC3946372

[BIO058843C74] Tsurumi, Y., Hamada, Y., Katoh, Y. and Nakayama, K. (2019). Interactions of the dynein-2 intermediate chain WDR34 with the light chains are required for ciliary retrograde protein trafficking. *Mol. Biol. Cell* 30, 658-670. 10.1091/mbc.E18-10-0678 30649997 PMC6589695

[BIO058843C75] Vakulskas, C. A., Dever, D. P., Rettig, G. R., Turk, R., Jacobi, A. M., Collingwood, M. A., Bode, N. M., McNeill, M. S., Yan, S., Camarena, J. et al. (2018). A high-fidelity Cas9 mutant delivered as a ribonucleoprotein complex enables efficient gene editing in human hematopoietic stem and progenitor cells. *Nat. Med.* 24, 1216-1224. 10.1038/s41591-018-0137-0 30082871 PMC6107069

[BIO058843C76] Van Valkenburgh, H., Shern, J. F., Sharer, J. D., Zhu, X. and Kahn, R. A. (2001). ADP-ribosylation factors (ARFs) and ARF-like 1 (ARL1) have both specific and shared effectors: characterizing ARF1-binding proteins. *J. Biol. Chem.* 276, 22826-22837. 10.1074/jbc.M102359200 11303027

[BIO058843C77] Wätzlich, D., Vetter, I., Gotthardt, K., Miertzschke, M., Chen, Y.-X., Wittinghofer, A. and Ismail, S. (2013). The interplay between RPGR, PDEδ and Arl2/3 regulate the ciliary targeting of farnesylated cargo. *EMBO Rep.* 14, 465-472. 10.1038/embor.2013.37 23559067 PMC3642377

[BIO058843C78] Yang, T. T., Su, J., Wang, W.-J., Craige, B., Witman, G. B., Tsou, M.-F. B. and Liao, J.-C. (2015). Superresolution pattern recognition reveals the architectural map of the ciliary transition zone. *Sci. Rep.* 5, 14096. 10.1038/srep14096 26365165 PMC4568515

[BIO058843C79] Ye, F., Nager, A. R. and Nachury, M. V. (2018). BBSome trains remove activated GPCRs from cilia by enabling passage through the transition zone. *J. Cell Biol.* 217, 1847-1868. 10.1083/jcb.201709041 29483145 PMC5940304

[BIO058843C80] Zhang, Q., Hu, J. and Ling, K. (2013). Molecular views of Arf-like small GTPases in cilia and ciliopathies. *Exp. Cell Res.* 319, 2316-2322. 10.1016/j.yexcr.2013.03.024 23548655 PMC3742637

[BIO058843C81] Zhang, Q., Li, Y., Zhang, Y., Torres, V. E., Harris, P. C., Ling, K. and Hu, J. (2016). GTP-binding of ARL-3 is activated by ARL-13 as a GEF and stabilized by UNC-119. *Sci. Rep.* 6, 24534. 10.1038/srep24534 27102355 PMC4840320

