# Supplementary material for: ARL3 and ARL13B GTPases participate in distinct steps of INPP5E targeting to the ciliary membrane
Source: Biol Open. 2021 Sep 28;10(9):bio058843. doi: 10.1242/bio.058843 (PMC8496693; doi:10.1242/bio.058843)
Supplement: Supplementary information [file biolopen-10-058843-s1.pdf]

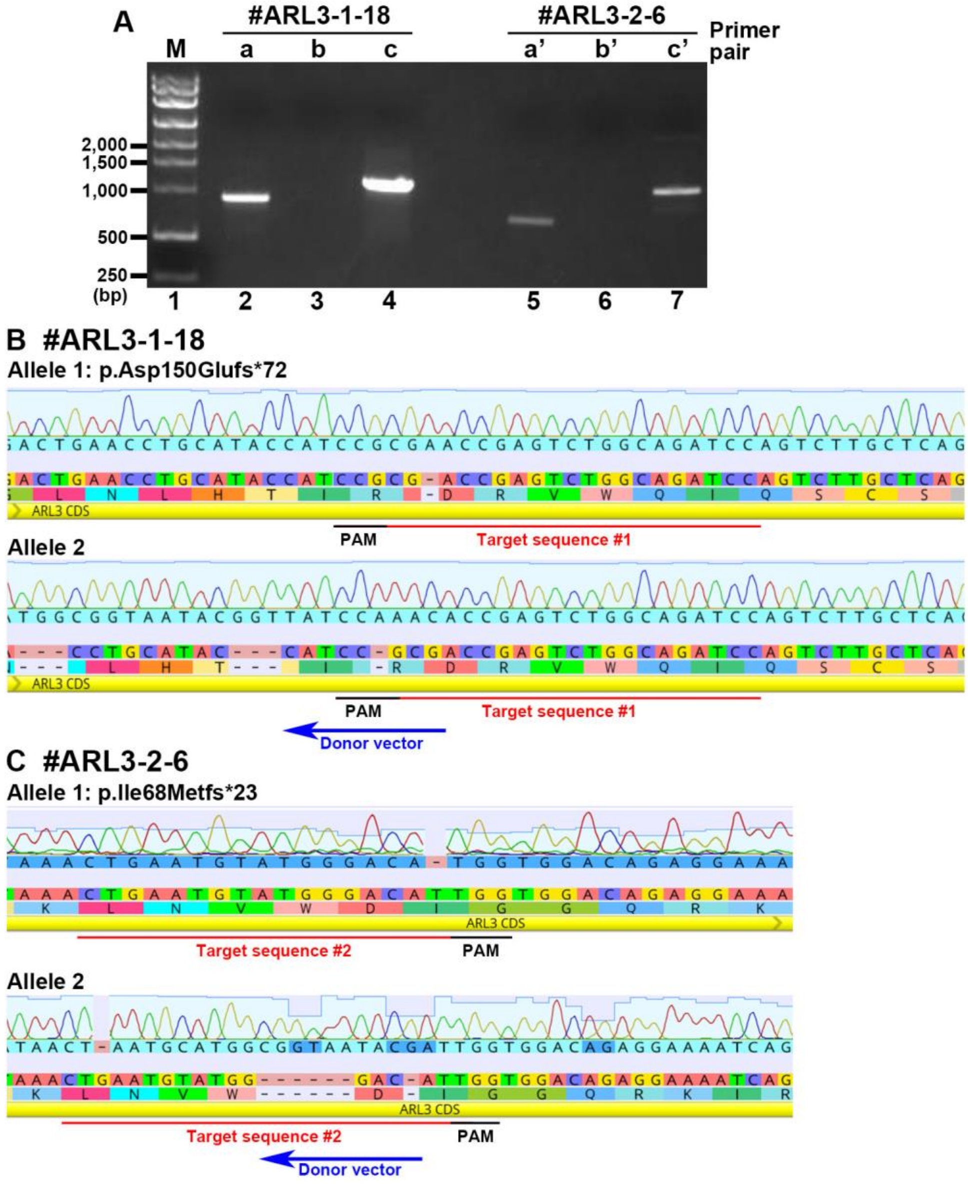

Fig. S1. Genomic PCR and sequence analyses of the *ARL3*-KO cell lines

(A) Genomic DNAs extracted from the *ARL3*-KO cell lines #ARL3-1-18 and #ARL3-2-6 were subjected to PCR using the indicated primer sets (see Table S3) to detect alleles with a small indel or no insertion (a, primers 1 + 2; a′, primers 4 + 5), or with forward (b, primers 1 + 3; b′, primers 4 + 3) or reverse (c, primers 2 + 3; c′, primers 5 + 3) integration of the donor knock-in vector. M, molecular weight marker (PSU1 DNA ladder). (B, C) Alignments of allele sequences of the # ARL3-1-18 (B) and # ARL3-2-6 (C) cell lines determined by sequencing of the PCR products shown in (A). Red and black lines indicate the target sequence and PAM sequence, respectively, and blue arrows indicate the direction of integration of the donor knock-in vector.

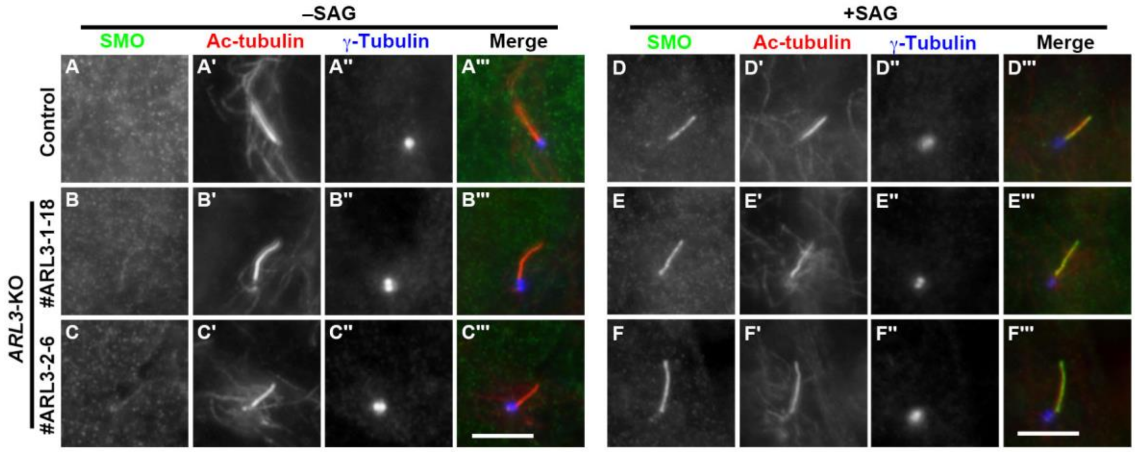

Fig. S2. Localization and trafficking of ciliary SMO are unaffected in *ARL3*-KO cells

Control RPE1 cells (A, D), and the *ARL3*-KO cell lines #ARL3-1-18 (B, E) and #ARL3-2-6 (C, F) were serum starved for 24 h, and then cultured in the absence (A–C; –SAG) or presence (D–F; +SAG) of 200 nM SAG for a further 24 h. The cells were then triply immunostained for SMO, Ac-tubulin, and γ-tubulin. Scale bar, 5 µm

Table S1. Plasmids used in this study

Vector	Insert	Reference
pCAG2-EGFP-C	INPP5E	(Nozaki et al., 2017)
pCAG2-EGFP-C	INPP5E (1–626)	This study
pCAG2-EGFP-C	INPP5E(ΔCTS)	(Qiu et al., 2021)
pCAG2-mCherry-C	INPP5E	(Nozaki et al., 2017)
pCAG2-mCherry-C	INPP5E (1–626)	This study
pCAG2-mCherry-C	INPP5E(ΔCTS)	(Qiu et al., 2021)
pCAG2-tBFP2-C	INPP5E	This study
pCAG-ds-EGFP-N	ARL13B	(Nozaki et al., 2017)
pCAG-ds-EGFP-N	ARL13B(T35N)	(Nozaki et al., 2017)
pCAG-ds-EGFP-N	ARL13B (R79Q)	(Nozaki et al., 2017)
pCAG-ds-EGFP-N	ARL13B (Y86C)	This study
pCAG-ds-EGFP-N	ARL13B (R200C)	(Nozaki et al., 2017)
pCAG-ds-EGFP-N	ARL13B (AAEA)	(Nozaki et al., 2017)
pCAG2-mCherry-N	ARL13B	(Nozaki et al., 2017)
pCAG-ds-tBFP2-N	ARL13B	This study
pRRLsiPPT-tRFP-T-N-IRES-Zeo	ARL13B	(Nozaki et al., 2017)
pRRLsiPPT-tRFP-T-N-IRES-Zeo	ARL13B(T35N)	(Nozaki et al., 2017)
pRRLsiPPT-tRFP-T-N-IRES-Zeo	ARL13B (R79Q)	(Nozaki et al., 2017)
pRRLsiPPT-tRFP-T-N-IRES-Zeo	ARL13B (Y86C)	This study
pRRLsiPPT-tRFP-T-N-IRES-Zeo	ARL13B (R200C)	(Nozaki et al., 2017)
pRRLsiPPT-tRFP-T-N-IRES-Zeo	ARL13B (AAEA)	(Nozaki et al., 2017)
pEGFP-N	ARL3	This study
pcDNA3-mCherry-N	ARL3	This study
pTagBFP2-N	ARL3	This study
pRRLsinPPT-EGFP-N--IRES-Zeo	ARL3	This study
pRRLsinPPT-EGFP-N--IRES-Zeo	ARL3 (QL)	This study
pRRLsinPPT-EGFP-N--IRES-Zeo	ARL3 (TN)	This study
pRRLsinPPT-EGFP-N--IRES-Zeo	ARL13B	This study
pEGFP-C1	PDE6D	This study
pmCherry-C1	PDE6D	This study
pTagBFP2-C	PDE6D	This study
pDonor-tBFP-NLS-Neo(universal)	—	(Katoh et al., 2017)
pHiFiCas9-2xsgRNA	—	This study
pGEX-6P1	Anti-GFP-nanobody	(Katoh et al., 2015)

Table S2. Antibodies used in this study

Antibody	Manufacturer	Clone/catalog number or reference number	Dilution (purpose)
Polyclonal rabbit anti-IFT88	Proteintech	13967-1-AP	1:500 (IF)
Polyclonal rabbit anti-IFT140	Proteintech	17460-1-AP	1:500 (IF)
Polyclonal rabbit anti-TULP3	Proteintech	136371-AP	1:200 (IF)
Polyclonal rabbit anti-INPP5E	Proteintech	17797-1-AP	1:500 (IF)
Polyclonal rabbit anti-GPR161	Proteintech	13398-1-AP	1:500 (IF)
Polyclonal rabbit anti-ARL6	Proteintech	12676-1-AP	1:500 (IF)
Polyclonal rabbit anti-BBS9	Atlas Antibodies	HPA021289	1:1,000 (IF)
Monoclonal mouse anti-ARL13B	Abcam	N295B/66	1:500 (IF)
Monoclonal mouse anti-SMO	Santa Cruz	sc-166685	1:100 (IF)
Monoclonal mouse anti-Ac-tubulin	Sigma-Aldrich	6-11B-1	1:1,000 (IF)
Monoclonal mouse anti-γ-tubulin	Sigma-Aldrich	GTU88	1:1,000 (IF)
Monoclonal mouse anti-FOP	Abnova	2B1	1:10,000 (IF)
Polyclonal rabbit anti-mCherry	Proteintech	26765-1-AP	1:5,000 (IB)
Monoclonal mouse anti-GFP	Proteintech	66002-1-Ig	1:10,000 (IB)
AlexaFluor-conjugated secondary	Molecular Probes	A11034, A21429, A21137, A21127, A21240, A21147, A21131, A21242	1:1,000 (IF)
Peroxidase-conjugated secondary	Jackson ImmunoResearch	115-035-166, 111-035-144	1:3,000 (IB)

IF, immunofluorescence; IB, immunoblotting.

Table S3. Oligo DNAs used in this study

Name	Sequence
ARL3 -genome-FW (primer 1)	5'- TCTGATTAGAACTGCATGGTTG-3'
ARL3 -genome-RV (primer 2)	5'- ACACAACACAAAACCCCAATC-3'
pTagBFP-N-RV2 (primer 3)	5'- CGTAGAGGAAGCTAGTAGCCAGG -3'
ARL3 -genome-FW (primer 4)	5'- TTACCATCCTGAAGATTCATGG -3'
ARL3 -genome-RV (primer 5)	5'- GATCTTCTGACTTCCCTTCTG -3'
ARL3-gRNA#1-S	5'- CACCGGATCTGCCAGACTCGGTCG-3'
ARL3-gRNA#1-AS	5'- AAACCGACCGAGTCTGGCAGATCC-3'
ARL3-gRNA#2-S	5'- CACCCTGAATGTATGGGACATTGG-3'
ARL3-gRNA#2-AS	5'- AAACCCAATGTCCCATACATTCAG-3'

FW, forward; RV, reverse; S, sense; AS, antisense.

Biology Open • Supplementary information
